# Integrated Multi-omics Analysis of Early Lung Adenocarcinoma Links Tumor Biological Features with Predicted Indolence or Aggressiveness

**DOI:** 10.1158/2767-9764.CRC-22-0373

**Published:** 2023-07-26

**Authors:** Maria-Fernanda Senosain, Yong Zou, Khushbu Patel, Shilin Zhao, Alexis Coullomb, Dianna J. Rowe, Jonathan M. Lehman, Jonathan M. Irish, Fabien Maldonado, Michael N. Kammer, Vera Pancaldi, Carlos F. Lopez

**Affiliations:** 1 Cancer Biology Graduate Program, Vanderbilt University, Nashville, Tennessee.; 2 Division of Allergy, Pulmonary, and Critical Care Medicine, Department of Medicine, Vanderbilt University Medical Center, Nashville, Tennessee.; 3 Cancer Early Detection and Prevention Initiative, Vanderbilt-Ingram Cancer Center, Vanderbilt University Medical. Center, Nashville, Tennessee.; 4 Vanderbilt Ingram Cancer Center, Department of Biostatistics, Vanderbilt University Medical Center, Nashville, Tennessee.; 5 CRCT, Université de Toulouse, Inserm, CNRS, Université Toulouse III-Paul Sabatier, Centre de Recherches en Cancérologie de Toulouse, Toulouse, France.; 6 Division of Hematology/Oncology, Department of Medicine, Vanderbilt University Medical Center, Nashville, Tennessee.; 7 Vanderbilt-Ingram Cancer Center, Vanderbilt University Medical Center, Nashville, Tennessee.; 8 Department of Cell and Developmental Biology, Vanderbilt University, Nashville, Tennessee.; 9 Department of Pathology, Microbiology and Immunology, Vanderbilt University Medical Center, Nashville, Tennessee.; 10 Barcelona Supercomputing Center, Carrer de Jordi Girona, 29, 31, 08034 Barcelona, Spain.; 11 Department of Biochemistry, Vanderbilt University, Nashville, Tennessee.; 12 Department of Biomedical Informatics, Vanderbilt University, Nashville, Tennessee.

## Abstract

Lung adenocarcinoma (LUAD) is a heterogeneous group of tumors associated with different survival rates, even when detected at an early stage. Here, we aim to investigate the biological determinants of early LUAD indolence or aggressiveness using radiomics as a surrogate of behavior. We present a set of 92 patients with LUAD with data collected across different methodologies. Patients were risk-stratified using the CT-based Score Indicative of Lung cancer Aggression (SILA) tool (0 = least aggressive, 1 = most aggressive). We grouped the patients as indolent (*x ≤* 0.4, *n* = 14), intermediate (0.4 *> x ≤* 0.6, *n* = 27), and aggressive (0.6 *> x ≤* 1, *n* = 52). Using Cytometry by time of flight (CyTOF), we identified subpopulations with high HLA-DR expression that were associated with indolent behavior. In the RNA sequencing (RNA-seq) dataset, pathways related to immune response were associated with indolent behavior, while pathways associated with cell cycle and proliferation were associated with aggressive behavior. We extracted quantitative radiomics features from the CT scans of the patients. Integrating these datasets, we identified four feature signatures and four patient clusters that were associated with survival. Using single-cell RNA-seq, we found that indolent tumors had significantly more T cells and less B cells than aggressive tumors, and that the latter had a higher abundance of regulatory T cells and Th cells. In conclusion, we were able to uncover a correspondence between radiomics and tumor biology, which could improve the discrimination between indolent and aggressive LUAD tumors, enhance our knowledge in the biology of these tumors, and offer novel and personalized avenues for intervention.

**Significance:**

This study provides a comprehensive profiling of LUAD indolence and aggressiveness at the biological bulk and single-cell levels, as well as at the clinical and radiomics levels. This hypothesis generating study uncovers several potential future research avenues. It also highlights the importance and power of data integration to improve our systemic understanding of LUAD and to help reduce the gap between basic science research and clinical practice.

## Introduction

Lung cancer has the highest mortality rate among cancers worldwide, causing more deaths than breast, cervical, prostate, and colorectal cancers, each of which have established population-based screening programs (1). The 5-year survival rate for patients with lung cancer is only 15%, mainly because 70% of them are diagnosed at a late stage (2). Among lung cancer subtypes, lung adenocarcinoma (LUAD) remains the most frequent (3). In the past years, the National Lung Screening Trial (NLST) and more recently the Nederlands–Leuvens Longkanker Screenings Onderzoek (NELSON) trial have shown that lung cancer mortality is significantly reduced in individuals who undergo low-dose and volume chest CT screening, respectively (4, 5). However, in both cases, the overdiagnosis rate for a follow-up of 10 years is relatively high, involving almost 1 out of 5 diagnosed patients (18.5% and 19.9%, respectively). In addition, LUAD is a heterogeneous disease both clinically and biologically. Recent advances in single-cell technologies have allowed researchers to dissect the cellular heterogeneity of tumors and learn more about the tumor microenvironment (TME) and its role in tumorigenesis, tumor development, progression, and metastasis (6–9). On the other hand, advances in imaging technologies, specifically in the radiomics field, have allowed for the development of new tools to aid diagnosis and prognosis of these tumors (10–16). Despite these research efforts, the biological determinants that account for the differences between aggressive and indolent tumors remain obscure. As previously described by Welch and Black (17), the trajectory of cancer progression can be widely heterogeneous, where the most aggressive and fast-growing tumors quickly lead to symptoms and death, and the most indolent or slow-growing tumors will never cause problems and the patient will die from other cause before the cancer is large enough to cause symptoms. Crucially, understanding the likely trajectory of the tumor has a direct implication in the efficacy and cost-effectiveness of lung cancer screening, particularly when considering the risks of overdiagnosis and overtreatment (18, 19). In a recent publication, we showed that we could dissect some of the main cell types of LUAD using single-cell technology and found that the protein expression of MHC-II was associated with indolent behavior and increased T-cell infiltration (20). Here, we investigate the biological determinants of early LUAD indolence or aggressiveness using radiomics as a surrogate of behavior. We hypothesize that integration of biological, clinical, and radiomics data of early-stage LUAD will improve the discrimination between indolent and aggressive tumors which in turn may offer novel and personalized avenues for intervention. To this end, we generated a unique and comprehensive multi-omics dataset and applied an integrative analytic strategy that provides a deep profiling of LUAD tumor biology. The integration of these molecular datasets with noninvasive CT-based risk stratification, suggests a potential mechanistic interpretation for widely used medical tools revealing in which way the biology of the tumor can be related to radiomics profiles.

## Materials and Methods

### Human Samples

Tumor tissues samples were collected from patients undergoing lung resection surgery following an Institutional Review Board–approved protocol 000616 at the Vanderbilt University Medical Center (Nashville, TN). Written informed consent was obtained from all subjects. Samples were obtained from 92 patients with LUAD, from which 43 were males and 49 were females. The ages from these patients ranged from 48 to 90 with a median of 66.5. See Supplementary Tables S1 and S2 for more details.

### Sample Collection and Processing

All tissue samples were processed within 1 hour of surgery. For single-cell suspension analyses, lung tissues were minced and digested with Collagenase and DNase I for 1 hour at 37°C. Single-cell suspension was sequentially filtered (70 and 40 μm) and cryopreserved for long-term storage as described previously (21). Cell viability was assessed before cryopreservation and after thawing. For bulk analyses, lung tissues were snap frozen and stored in liquid nitrogen.

### Patient Risk Stratification and Radiomics

#### Score Indicative of Lung Cancer Aggression

Score Indicative of Lung Cancer Aggression (SILA) is a cumulative aggregate of normalized distributions of the ordered CANARY exemplars (10) and provides a continuous variable in range of 0 to 1 (14). In addition to discrimination between indolent and invasive adenocarcinoma, it also helps in predicting the degree of invasion, disease-free survival, and cancer-related mortality in stage I LUAD on the basis of CT. The continuous scale can be thresholded at multiple levels, if needed. We set two SILA thresholds and categorized three distinct histopathologic and prognostic groups for stage I LUAD. These thresholds were computed by using two approaches: automatic histogram-based multilevel thresholding and pathology-based threshold selection. In the automatic approach, the histogram constructed from the SILA values for stage I LUAD nodules in the cohort is divided into three partitions by using a well-known multilevel thresholding algorithm. Pathology-based SILA thresholds were assigned on the basis of TImax (maximum linear extent of tumor invasion) in stage I LUAD. Three distinct survival groups were discovered: best survival in indolent tumors (AIS and MIA), intermediate survival in tumors with TImax from 6 to 20 mm, and worst survival in tumors with TImax greater than 20 mm. The group with a SILA of 0.338 or lower [SILA at the upper 95% confidence interval (CI) of the indolent group] was defined as the good-prognosis group. The group with a SILA of 0.338 to 0.675 (SILA at the lower 95% CI of the aggressive group) was defined as the intermediate prognosis group, and the group with a SILA of 0.675 or higher was defined as the poor-prognosis group.

#### HealthMyne

HealthMyne platform allows semiautomatic lesion segmentation of the delineated volumes of interest, followed by extraction of radiomics features. The user initializes the lesion segmentation by drawing a long axis on region of interest in an axial plane of the multiplanar reconstruction (MPR). A two-dimensional (2D) segmentation is updated in real time with interactive feedback of the lesion boundary and 2D segmentations on the other MPR planes are immediately proposed. If the contour on a MPR plane seems unsatisfactory, the user can update the segmentation by either drawing long axes on the other MPR views or using a 2D brush tool. When the segmentation is satisfactory, the user can confirm to initiate the three-dimensional (3D) segmentation computation. On the basis of these initial user interactions, the reads per million (RPM) algorithms combined statistical sampling methods together with deep learning strategies to delineate the target volume and provide an automatic 3D segmentation. The 3D segmentation is reviewed by scrolling through slices on the MPR views. Interactive editing tools including 2D and 3D brushes can be used to reduce/enlarge or add details to the proposed volume segmentation. As the 3D segmentation is confirmed by the user, the measure of the long and short lesion axes is automatically determined by leveraging the volume delineation. Many radiomics features are extracted from the segmented volume. Redundant features or features with high interuser/intrauser variability were removed. The radiomics risk score is derived from regression shrinkage and subset selection via Least Absolute Shrinkage and Selection Operator (LASSO) method.

### Mass Cytometry

#### Antibody Panel

We have developed a comprehensive antibody panel that comprises a total of 34 antibodies, including markers for cellular lineage (immune cells, epithelial cells, endothelial cells, fibroblasts/mesenchymal cells), cancer markers, and signaling pathways. Metal-conjugated antibodies were purchased from Fluidigm and customized conjugations were performed using Maxpar Multi-Metal labeling Kits (Fluidigm) with purified antibodies from different sources (see Supplementary Table S4).

#### Sample Preparation and Data Acquisition

Cryopreserved samples were thawed and stained with our antibody panel (Supplementary Table S4) as described previously (21). For intracellular staining, cells were permeabilized with methanol. To prevent cell loss, an additional fixation step was added to the protocol after the washing steps of the intracellular staining. We controlled for batch effect using EQ Four Element Calibration Beads (DVS Sciences/Fluidigm). Prior sample acquisition, cells were resuspended in 1X calibration beads in deionized water to reach a concentration of 5 *×* 10^5^ cells/mL. Cells were filtered using FACS tubes with filter caps (Corning Falcon) and collected using a standard/narrow bore on a Helios CyTOF system at the Mass Cytometry Center of Excellence at Vanderbilt University.

#### Data Preprocessing

Prior to analysis, all mass cytometry FCS files were normalized using the premessa R package (https://github.com/ParkerICI/premessa, version 0.2.4), an R implementation of the MATLAB bead normalization software (22). Noise reduction parameters were same a described previously (20).

#### Data Analysis

To determine cellular identity, we performed *k*-means using markers that identify main cellular populations [EpCAM, CD31, CD45, vimentin, cytokeratin, and cytokeratin 7 (CK7)]. The optimal number of clusters was determined by calculating the within cluster sum of squares (WSS) for different *k* values, plotting *k* versus WSS and choosing the *k* in which we see a pronounced bend or “elbow” (*k* = 10). The clusters were annotated on the basis of protein expression and clusters with similar characteristics were merged. Final cell types were annotated as epithelial cancer cells (ECC; EpCAM+/cytokeratin+/cytokeratin7+), endothelial cells (CD45^−^/CD31^+^), fibroblasts/mesenchymal cells (vimentin+/CD45^−^/CD31^−^/EpCAM−/cytokeratin−/cytokeratin7−), and immune cells (CD45^+^). We performed a second clustering round for immune cells only using immune cell markers CD8, CD4, CD3, CD11b, and CD56. Clusters were annotated into myeloid cells (CD45^+^/CD3^−^/CD11b^+^), CD8^+^ T cells (CD45^+^/CD3^+^/CD8^+^), CD4^+^ T cells (CD45^+^/CD3^+^/CD4^+^), double-negative T cells (CD45^+^/CD3^+^/CD4^−^/CD8^−^), and other immune as the remaining CD45^+^ cells. Each identified cell subset, including the nonimmune cells, underwent an independent round of clustering using the protein markers showed in their corresponding heat map (Supplementary Fig. S2C–S9C). We then calculated the percentage of each subset per patient and compared cluster frequencies between groups using nonparametric Wilcoxon rank-sum test (Supplementary Fig. S2D–S9D). For each cell type, we calculated the Spearman correlation between protein markers (Supplementary Fig. S2E–S9E). We also calculated the Spearman correlation of the proportion of cell type clusters among the patients (Supplementary Fig. S10). Finally, we calculated the bulk median protein per patient and compared patients between groups using nonparametric Wilcoxon rank-sum test (Supplementary Fig. S11).

### Whole-exome Sequencing

#### Sample Preparation and Data Acquisition

DNA was extracted using the DNeasy Blood & Tissue Kit (Qiagen) following the kit protocol. A quantitation and integrity assessment were completed using the whole-genomic DNA. An aliquot of each sample was analyzed on the Agilent TapeStation and quantified using a Picogreen assay. The samples were normalized and plated using the BioMek FX liquid handler. Libraries were prepared using 12–50 ng of DNA and the Twist Biosciences library preparation kit (P/N 104207) per manufacturer's instructions. Libraries were then captured using the Twist Comprehensive Exome panel (P/N 102031). Individual libraries were assessed for quality using the Agilent 2100 Bioanalyzer and quantified with a Qubit Fluorometer. The adapter ligated material was evaluated using qPCR prior to normalization and pooling for sequencing on the QuantStudio 12K Flex. The libraries were sequenced using the NovaSeq 6000 instrument with 150 bp paired end reads. RTA (version 2.4.11; Illumina) was used for base calling and data quality control (QC) was completed using MultiQC v1.7. Each sample was analyzed using the DRAGEN Enrichment Pipeline v3.7.5 to calculate alignment and capture metrics.

#### Data Preprocessing

Sequence data from genomic DNA were aligned to the reference human genome (GRCh38) by Burrows-Wheeler Aligner aligner (23). For QC purpose, multiple stages of QC on sequencing data were carried out. Raw data QC was performed by FastQC (24) and QC3 (25). Alignment QC and Variants QC were performed using QC3 (25). GATK software 4.1.8.1 was used for somatic single-nucleotide variants (SNV), short insertion and deletion variant (INDEL), and somatic copy-number variation (CNV) calling (26). Briefly, the reads preprocessing (RealignerTargetCreator, IndelRealigner, BaseRecalibrator) was performed as described in GATK Best Practices Workflows (26). Then MuTect2 (27) was used for somatic mutation (SNVs and INDELs) calling and GATK was used for somatic CNV calling. All the identified variants were annotated by ANNOVAR to gene and transcript level (28). All variants outside the target regions or synonymous variants were removed. Then all the variants were annotated to public database including dbSNP (29), Exome Aggregation Consortium (ExAC; ref. 30), NHLBI GO Exome Sequencing Project (ESP), and COSMIC (31). To remove possible germline mutations, variants reported in dbSNp or ExAC or ESP with minor allele frequency in normal population larger than 1% were removed.

#### Data Analysis

The resulting processed file (Mutation Annotation Format, MAF) was analyzed using the R package maftools (32). We used the Oncoplot to visualize the top 25 mutated genes, and the Forest plot to compare indolent + intermediate tumors versus aggressive and identify the significantly mutated genes (Supplementary Fig. S12A and S12C). Finally, we calculated the Spearman correlation between the SILA score and the logarithm base 10 of the mutational load (number of mutations per patients; Supplementary Fig. S12B).

### Bulk RNA-seq

#### Sample Preparation and Data Acquisition

RNA was extracted using the RNeasy Plus Mini Kit (Qiagen) following the kit protocol. RNA-seq libraries were prepared using 300 ng of RNA and the NEBNext Ultra II Directional RNA Library Prep kit (NEB, catalog no.: E7760L). Fragmentation, cDNA synthesis, end repair/dA-tailing, adaptor ligation and PCR enrichment were performed per manufacturer's instructions. Individual libraries were assessed for quality using the Agilent 2100 Bioanalyzer and quantified with a Qubit Fluorometer. The adapter ligated material was evaluated using qPCR prior to normalization and pooling for sequencing. The libraries were sequenced using the NovaSeq 6000 with 150 bp paired end reads. RTA (version 2.4.11; Illumina) was used for base calling and data QC was completed using MultiQC v1.7 by the Vanderbilt Technologies for Advanced Genomics (VANTAGE) core (Vanderbilt University, Nashville, TN).

#### Data Preprocessing

QC analysis was performed on all sequencing reads using FastQC package developed by the Babraham Institute bioinformatics group. Reads with poor quality were trimmed and adapter sequences were removed by cutadapt g. Reads were then aligned to human genome (hg38) using STAR (33) and quantified by featureCounts (34). Alignment quality was checked by QC3 (25). Any RNA-seq experiment with poor quality was removed.

#### Data Analysis

Starting from the raw counts, we removed low variance genes and filtered out genes from chromosomes X and Y. We used the package DESeq2 to perform differential gene expression analysis (35) and the package fgsea for the gene set enrichment analyses (GSEA; ref. 36) with the Molecular Signature Database (MSigDB) hallmark gene set collection (37) and the REACTOME database (38). The transcription factor (TF) activity was inferred using the VIPER package (39). Individual pathways scores per patient sample were obtained using the Gene Set Variation Analysis (GSVA) tool (40).

### Single-cell RNA-seq

#### Sample Preparation and Data Acquisition

After dead cell removal with MACS Dead Cell Removal Kit, (Miltenyi Biotec), cells (5,000–10,000 cells per sample) were submitted for processing using the 10X Genomics platform. Libraries were prepared using P/N 1000006, 1000080, and 1000020 following the manufacturer's protocol. The libraries were sequenced using the NovaSeq 6000 with 150 bp paired end reads. RTA (version 2.4.11; Illumina) was used for base calling and analysis was completed using 10X Genomics Cell Ranger software v4.0.0.

#### Data Preprocessing

We used 10x Genomics Cell Ranger 4.0.0 software to obtain the feature barcode matrices per sample. For further preprocessing steps, we used the scanpy tool (41). For more details, see https://scanpy-tutorials.readthedocs.io/en/latest/pbmc3k.html.

#### Data Analysis

We first computed a principal component analysis (PCA) to reduce the dimensionality of the data and then computed a neighborhood graph on the first 40 principal components. We then used the Leiden graph-clustering method (42) and obtained 25 clusters which then were annotated into seven major cell types: B cells, cancer cells, endothelial cells, mural cells, myeloid cells, and T cells. We calculated the cell type proportions for each patient and compared indolent versus aggressive tumors using nonparametric Wilcoxon rank-sum test. Each cell type underwent an additional clustering step and again cluster proportions between groups were compared. To better understand the identity of the clusters, we used the split violin visualization from scanpy and showed the top 30 marker genes for each cluster when compared with the rest.

### Data Integration

For the data integration effort, we selected only the features that were significantly associated with tumor behavior. From the CyTOF dataset, we included the cell type cluster proportions and the bulk protein expression per patient. In the latter, for a protein marker to be considered, the median of at least one patient group (indolent, intermediate, or aggressive) should be above 1.44, which in raw values (before the arcsinh transform) correspond to 10 “pushes” which is the default lower limit of the Helios (43). From the RNA-seq dataset, we selected all the pathways with *P*_adjusted_ value < 0.05, and a normalized enrichment score >1.5. We then used the GSVA package to calculate individual expression scores of these pathways for each patient. For the HealthMyne radiomics features dataset, we performed a Spearman pairwise correlation against the SILA score and selected only those significantly correlated (*P*_adjusted_ value < 0.05). Only patients with complete data were selected, all the matrices concatenated, and the features were scaled and centered. Patients and features were clustered independently using *k* means (*k* = 4, by elbow method as described in the CyTOF methods section). Cluster IDs for each patient and feature can be found in Supplementary Tables S11 and S12. To visualize the feature interactions, we computed a similarity matrix and also performed a PCA and plotted the first two components for both features and patients.

### Additional Statistical Analyses

For correlation analysis, we used Spearman rank correlation test and adjusted *P* values for multiple hypothesis using the Benjamini and Hochberg method (44). Comparison of categorical variables was performed using the Wilcoxon rank-sum test. Survival curves were generated using the Kaplan–Meier method, and statistically significant differences were analyzed with the log-rank test. All statistical tests were two sided and *P* values less than 0.05 were considered statistically significant. The analyses were performed in R 4.0.3 and Python 3.

### Code and Data Availability

All the code used to analyze the data and generate the visualizations and tables can be accessed at https://github.com/msenosain/TMA36_data-analysis. All processed data can be accessed at https://doi.org/10.5281/zenodo.7878082. Further information and requests for resources and raw data should be directed to and will be provided by Fabien Maldonado (fabien.maldonado@vumc.org). All methods were carried out in accordance with relevant guidelines and regulations.

## Results

### Multi-omic Profiling of LUAD Tumors Using Radiomics as a Surrogate of Behavior

To characterize the biological landscape of LUAD in association with their radiomics-based predicted behavior (i.e., indolent vs. aggressive), we designed a multi-omic profiling study of surgically removed primary tumors. We present a comprehensive set of 92 patients with LUAD who were treatment naïve at the time of surgery and were representative of the LUAD distribution across age, sex, mutational status, and smoking status (Supplementary Tables S1 and S2). In addition, over 90% of the cohort is composed of early-stage tumors (i.e., stages I–II; ref. 45).

**FIGURE 1:**
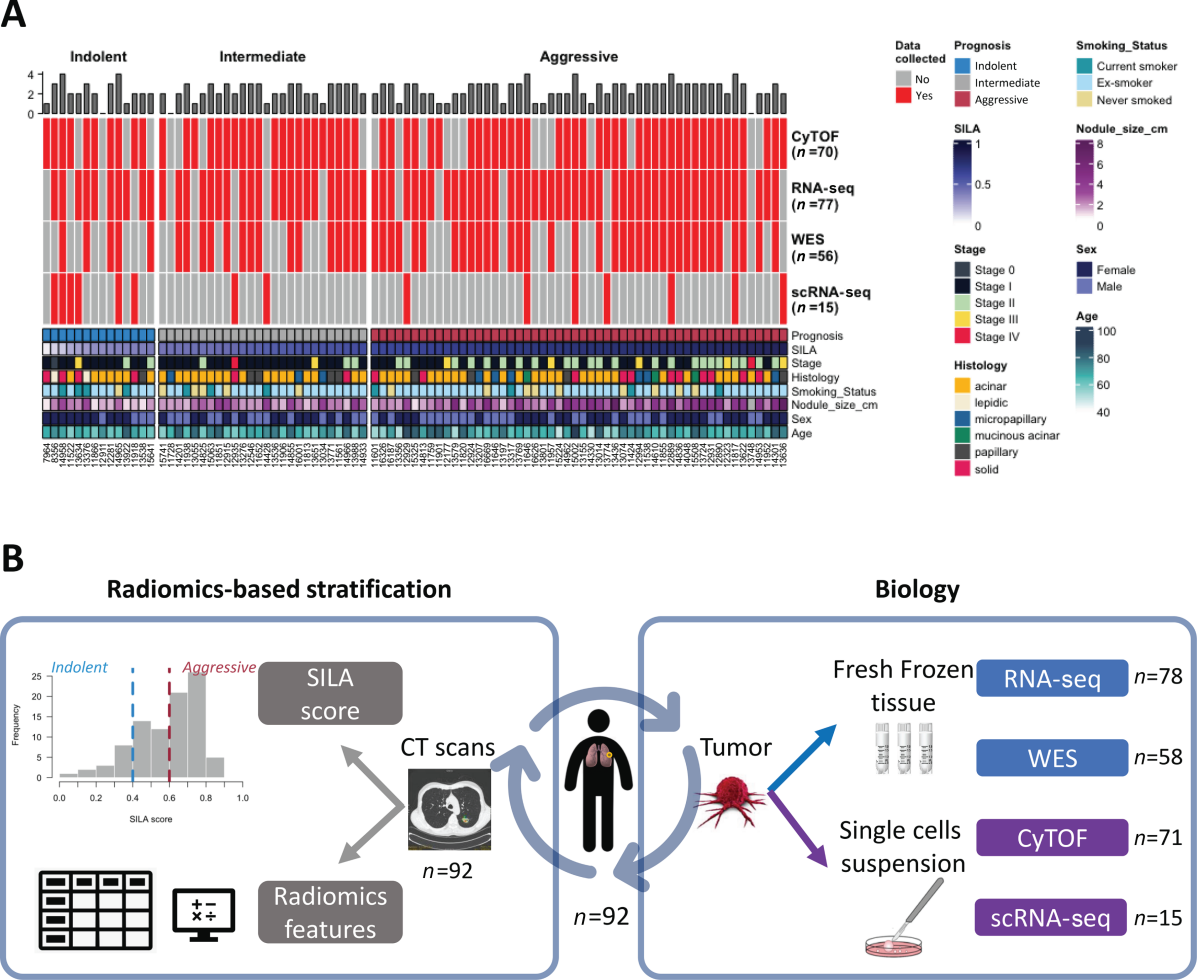
Summary of LUAD datasets and study workflow. **A,** Heat map showing the tissue-based datasets included in this study (rows) by patient (columns) where red means data have been collected for that specific patient and gray that it has not. The bars above the heat map correspond to the number of datasets collected for each patient. The bottom annotation shows some clinical characteristics of the patient cohort. **B,** Study workflow. Left, For each patient (*n* = 92), the CT scan taken closest to the day of surgery was retrieved. These were used (i) to calculate the SILA score and stratify the patients; and (ii) to extract a numeric matrix with their radiomics features using the HealthMyne software. Right, When tissue was available for these patients, as described in A, from fresh frozen tissue we collected bulk datasets (in blue) such as RNA-seq and WES, and from single-cell suspension we collected single-cell datasets (in purple) such as CyTOF and single-cell RNA-seq.

Data were collected using different assays (Fig. 1; Supplementary Table S3). Surgically removed specimens (one per patient) were split and processed as: single-cell suspension for CyTOF and single-cell RNA-seq, and fresh frozen tissue for RNA-seq and whole-exome sequencing (WES). Although data collection at every level was not possible for all specimens, there is some overlap in most datasets, allowing data integration (Fig. 1A).

In addition to the clinical data, chest CT scans for each patient taken within 3 months prior to surgery were analyzed and radiomics features were extracted with the HealthMyne software (12). To risk-stratify the patients, we used the CT-based SILA which analyses the CT scans of the patients and outputs a continuous score that ranges between 0 and 1, 0 being the least aggressive and 1 the most aggressive. This score has been validated to accurately correlate with histopathologic assessment, providing a scoring system to noninvasively predict the degree of histologic tumor invasion in LUAD (14). We then grouped these into three classes: indolent (*x ≤* 0.4, *n* = 14), intermediate (0.4 *> x ≤* 0.6, *n* = 27), and aggressive (0.6 *> x ≤* 1, *n* = 52; Fig. 1B, left).

### LUADs of Predicted Indolent Behavior are Enriched in HLA-DR Protein Expression

**FIGURE 2:**
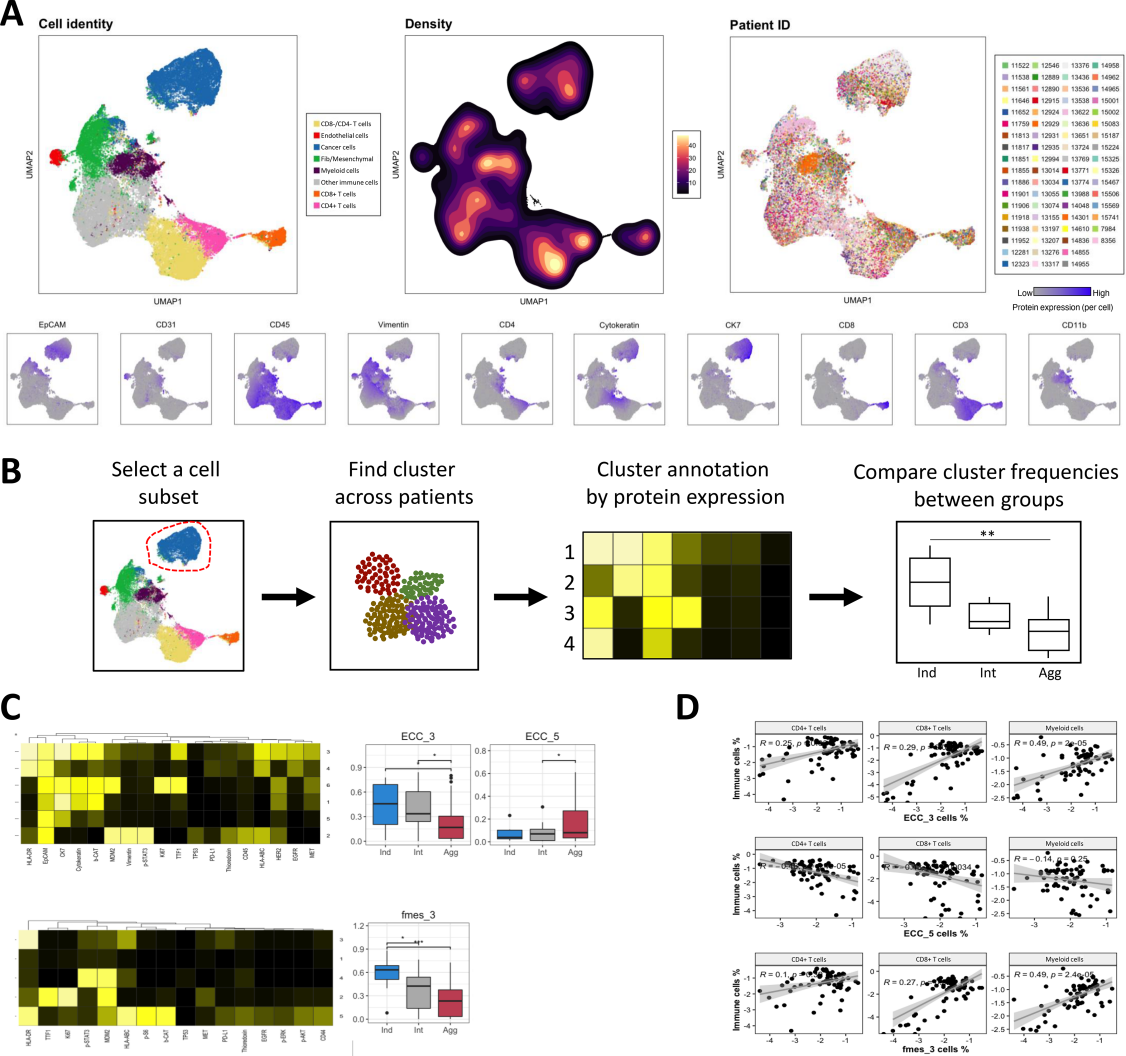
CyTOF analysis of LUAD samples reveal subsets associated with HLA-DR protein expression. **A,** Uniform Manifold Approximation and Projection (UMAP) representation colored by cell type [epithelial cancer cells (blue), endothelial cells (red), fibroblasts/mesenchymal cells (green), CD8^+^ T cells (orange), CD4^+^ T cells (pink), double-negative T cells (yellow), myeloid cells (purple), and other immune cells (gray)], by density, by patient ID, and by protein expression of lineage markers (bottom). **B,** Analysis workflow of the clustering by cell subset. **C,** Heat map of median protein expression per protein marker per cluster (left) and differential abundance analysis (right) for ECC (top) and fibroblast/mesenchymal cells (bottom). *Y* axis corresponds to the fraction of cells per patient sample. No star, *P* > 0.05; *, *P* < 0.05; **, *P* < 0.01; ***, *P* < 0.001. **D,** Spearman correlation analysis of the relative abundance of ECC3, 5 and Fmes 3 versus CD4^+^, CD8^+^ T cells, and myeloid cells, respectively.

LUADs human samples characterized by different predicted behavior classified into indolent (*n* = 10), intermediate (*n* = 21), and aggressive (*n* = 39) were stained with our previously validated antibody panel (20). We identified the major cell types (ECCs, endothelial cells, mesenchymal cells, and immune cells) based on the expression of protein markers (Fig. 2A). EpCAM+/pan cytokeratin+/cytokeratin 7+ cells were annotated as ECC; CD31^+^/CD45^−^ cells were annotated as endothelial cells; vimentin+/CD31^−^/CD45^−^ and negative for epithelial markers cells were annotated as mesenchymal cells. All CD45^+^ cells were annotated as immune cells. The latter were further classified into CD4^+^ T cells (CD3^+^/CD4^+^/CD8^−^), CD8^+^ T cells (CD3^+^/CD8^+^/CD4^−^), double-negative T cells (CD3^+^/CD8^−^/CD4^−^), myeloid cells (CD11b^+^/CD3^−^), and the remainder CD45^+^ cells were annotated as “Other Immune.” The relative abundance (frequencies) of these main cell types was not significantly different between patient groups (Supplementary Fig. S1).

Each subset individually went through an additional clustering step. Clusters were annotated by protein expression and then their frequencies within individual patient samples were compared between groups (Fig. 2B). In the ECC compartment, from a total of six clusters ECC cluster 3 (ECC3) relative abundance was significantly higher in patients with predicted indolent and intermediate behavior compared with aggressive (Fig. 2C; Supplementary Fig. S2). ECC3 is characterized by a high expression of HLA-DR, pan-cytokeratin, CK7, *beta*-catenin, and TTF1, as opposed to ECC4 which is other ECC cluster that expresses HLA-DR but lacks expression of all the other proteins mentioned. ECC5 and ECC2 were significantly higher in aggressive LUAD compared with intermediate; however, the latter was mainly composed by two tumors only. The former lacked expression of every other marker except for EpCAM and CK7, whereas the latter presented high expression of EpCAM, vimentin, MDM2, and p-STAT3. ECC6 was the only cluster expressing the proliferation marker Ki67, with aggressive tumors having a slightly higher median compared with the other groups. In terms of protein coexpression, HLA-DR, HLA-ABC, and EpCAM protein expression were highly correlated (*r >* 0.45, *P <* 0.05), and PD-L1 expression was also correlated with the first two (*r >* 0.4, *P <* 0.05; Supplementary Fig. S2E). Another group of highly correlated proteins were pan-cytokeratin, CK7, and *beta*-catenin, as well as the pairs of MET and EGFR, and TTF1 and Ki67 (*r >* 0.45, *P <* 0.05; Supplementary Fig. S2E). In the fibroblasts/mesenchymal cells compartment cluster 3 (Fmes3) relative abundance was significantly higher in patients with predicted indolent and intermediate behavior compared with aggressive (Fig. 2C; Supplementary Fig. S4). Fmes3 presented the highest expression of HLA-DR among the five clusters and had a moderate expression of HLA-ABC. This cell type also presented a subset engaged in proliferation (Fmes2) with high expression of Ki67, TTF1, and MDM2 (Supplementary Fig. S2C). In the protein coexpression analysis, Ki67 and TTF1 showed the highest correlation (*r* = 0.65), followed by p-STAT3 and MDM2 (*r* = 0.47), and HLA-DR and PD-L1 (*r* = 0.45; Supplementary Fig. S4E). HLA-DR and HLA-ABC correlation was also significant but not as high as in the cancer cells (*r* = 0.38). Although our CyTOF panel did not include sufficient markers to further annotate the identified immune cell types, we also performed reclustering on these with the aim of uncovering some degree of heterogeneity if present (e.g., proliferative vs. nonproliferative; Supplementary Fig. S5–S9). OIC cluster 4 (OIC4) was significantly enriched in patients with predicted indolent behavior compared with aggressive, and it was characterized by a high HLA-DR, HLA-ABC, and vimentin expression (Supplementary Fig. S9). OIC2 was significantly enriched in aggressive compared with indolent tumors, and it was characterized for the lack of expression of most markers and a moderate to low vimentin expression. Furthermore, the expression of HLA-DR and HLA-ABC was highly correlated (*r* = 0.61, *P <* 0.05), as was the expression of Ki67, TTF1, and MDM2 (*r >* 0.45, *P <* 0.05). In addition, as HLA-DR (an isotype of MHC-II) is known to be involved in antigen presentation, we wanted to see whether the relative abundance of the above-mentioned subsets were significantly correlated with enrichment or depletion of CD8^+^ and CD4^+^ T cells and myeloid cells (Fig. 2D; Supplementary Fig. S10). Indeed, ECC3, fmes3, and OIC4, clusters enriched in indolent tumors, were positively correlated with CD8^+^ and CD4^+^ T cells and myeloid cells, whereas ECC5 and OIC2, clusters enriched in aggressive tumors were negatively correlated with CD8^+^ and CD4^+^ T cells and myeloid cells. Finally, we calculated the median “bulk” protein expression for each protein per sample (Supplementary Fig. S11). We found that bulk HLA-DR protein expression is significantly higher in indolent and intermediate tumors compared with aggressive. Altogether, these results validate our previous findings (20), showing that HLA-DR expression in cancer cells and now also in fibroblasts/mesenchymal cells correlates with T-cell and myeloid cell enrichment and that these cells are particularly abundant in LUADs with indolent behavior, calling for a potentially immunogenic environment and therefore a more favorable prognosis.

### Transcriptomic Profiles of LUADs are Associated with Proliferation, Immune Response, and Extracellular Matrix Organization

Fresh frozen tissue from a set of 77 LUADs human samples characterized by different predicted behavior (indolent *n* = 10, intermediate *n* = 21, aggressive *n* = 46) was processed and the RNA was extracted and sequenced. A subset of those were also used to obtain WES (indolent *n* = 5, intermediate *n* = 15, aggressive *n* = 36) for genomic analysis. The mutational landscape of our LUAD cohort was very similar to what is expected for this cancer type (46, 47), with *KRAS* being the top mutated gene (41%) followed by *RYR2* (34%) and *MUC16* (32%; Supplementary Fig. S12A). *TP53* (27%) and *EGFR* (21%) were also among the top 15 mutated genes, and the latter was exclusive from *KRAS* alterations, as expected. We computed the mutational load for all 56 samples and found that it was mildly but significantly correlated with the SILA score (*r* = 0.27, *P* = 0.04), suggesting that genomic instability increases with the degree of predicted aggressiveness of the tumor (Supplementary Fig. S12B). To perform a clinical enrichment analysis of the mutations, we opted for combining indolent and intermediate tumors, as the former was too small to compare on its own. Among the top significantly enriched tumors in aggressive samples versus the indolent+intermediate group were *CTNND2*, *CACNA1E*, *SORCS1*, *PRDM9*, *NPAP1*, *APOB,* and *ADAMTS12* (Supplementary Fig. S12C).

**FIGURE 3:**
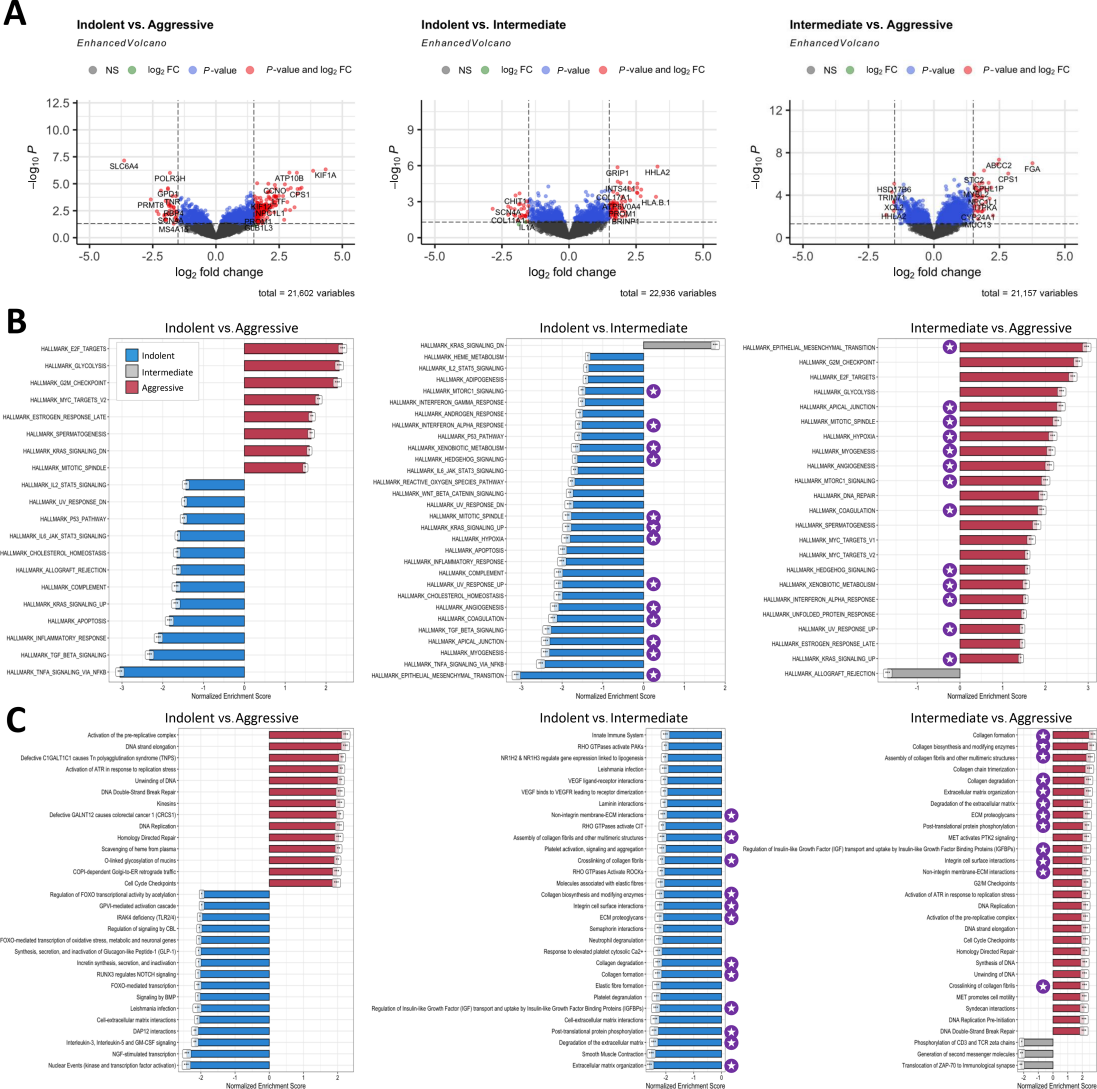
Transcriptomic analysis of LUAD highlights profiles associated with risk stratification. **A,** Volcano plots for indolent versus aggressive, indolent versus intermediate, and intermediate versus aggressive tumors, showing DEGs by fold change (FC) and *P* value. Cutoffs are log_2_FC> |1.5| and *P* < 0.05 GSEA with Hallmark (**B**) and REACTOME (**C**) databases for indolent versus aggressive, indolent versus intermediate, and intermediate versus aggressive tumors. Purple icon indicates pathways upregulated in both indolent and aggressive tumors when compared with intermediate.

We then performed differential gene expression analysis on the RNA-seq data (Fig. 3A; Supplementary Table S5). When comparing indolent versus aggressive, among the top dysregulated genes were *SLC6A4*, *KIF1A*, *HMGA2*, *ATP10B*, *POLR3H*, *GRIP1*, and *INTS4L1*. When comparing indolent versus intermediate, some of the top dysregulated genes were *HHLA2*, *GRIP1*, *DLGAP1-AS5*, *INTS4L1*, *PKHD1*, and *IGHV4-61*. When comparing intermediate versus aggressive, the top dysregulated genes were *ABCC2*, *FGA*, *B4GALNT1*, *MEGF10*, *CPS1*, and *STC2*. A detailed list of the differentially expressed genes (DEG) is presented in Supplementary Table S5. Furthermore, GSEA of the DEGs was performed to understand their biological functions in the patient groups with different predicted behavior using the Hallmark (37) and REACTOME (38) databases (Fig. 3B and C; Supplementary Tables S7–S9). When comparing aggressive versus indolent or aggressive versus intermediate, pathways associated with proliferation and cell cycle were upregulated, such as G_2_–M checkpoint, *E2F* targets, DNA replication, and elongation, etc. This suggests that the tumors predicted to be aggressive, share a strong proliferative signal compared with tumors with lower SILA scores. On the other hand, when comparing indolent versus aggressive or indolent versus intermediate, pathways related with immune response were upregulated, such as Inflammatory response, Complement, *TGF-beta* signaling, *TNF-alpha* signaling via *NFkB*, Leishmania infection, *IL3*, *IL5,* and *GMCSF*, Innate immune system, etc. Even though we saw the “Allograft rejection” pathway (a pathway associated with the expression of MHC classes I and II genes) present when comparing indolent or intermediate versus aggressive tumors, the pathways “Antigen processing-Cross presentation” and “MHC class II antigen presentation” were only upregulated in aggressive when compared with intermediate, suggesting that the high HLA-DR protein expression we previously saw associated with indolent tumors (Fig. 2C and D) might be a consequence of an inflammatory microenvironment rather than the cause of inflammation by antigen presentation. Interestingly, when comparing either aggressive or indolent versus intermediate, pathways related to structural components such as extracellular matrix (ECM) organization, collagen formation or degradation, epithelial–mesenchymal transition (EMT), angiogenesis, hypoxia, among others, were upregulated. A detailed list of the dysregulated pathways is presented in Supplementary Tables S7–S9. Finally, we used the VIPER algorithm to infer TF activity from gene expression data in the compared groups (Supplementary Table S6). When comparing indolent versus aggressive gene expression, the *FOXO1* and *SPI1* regulons were downregulated in aggressive tumors; when comparing indolent versus intermediate, the *HIF1A* and *SPI1* regulons were downregulated in intermediate tumors; and when comparing intermediate versus aggressive, the *FOXM1* and *HIF1A* regulons were upregulated in aggressive tumors. We see once again a pattern shared by indolent and aggressive tumors, this time the activation of the *HIF1A* regulon, which correlates well with the structural pathways upregulated in these patients.

### Data Integration Reveals an Association Between Radiomics Features and Tumor Biology

A fundamental part of this study is the use of computer-extracted quantitative features from the chest CT scans of the patients with LUAD, also known as radiomics. We first used SILA to obtain a score predictive of tumor aggressiveness and risk-stratify our cohort (Fig. 1). However, we are also interested in dissecting these images at a more granular level. Using the HealthMyne picture archiving and communication system (www.healthmyne.com), lung nodules were segmented from CT scans for feature extraction (12). While SILA informed us about the predicted aggressiveness of the tumor, HealthMyne provides information about the shape, form, and texture of the tumor with terminology commonly used in the clinical setting, providing a link between basic science and clinical interpretation. We obtained 300+ features, and then we filtered those that were significantly correlated with the SILA score (*P <* 0.05). We then ended up with 61 features, and only five of them were negatively correlated with the SILA score (i.e., features associated with good prognosis; Supplementary Table S10). Percentage of ground glass opacity was one of them, whereas solid percentage was positively correlated with SILA.

**FIGURE 4:**
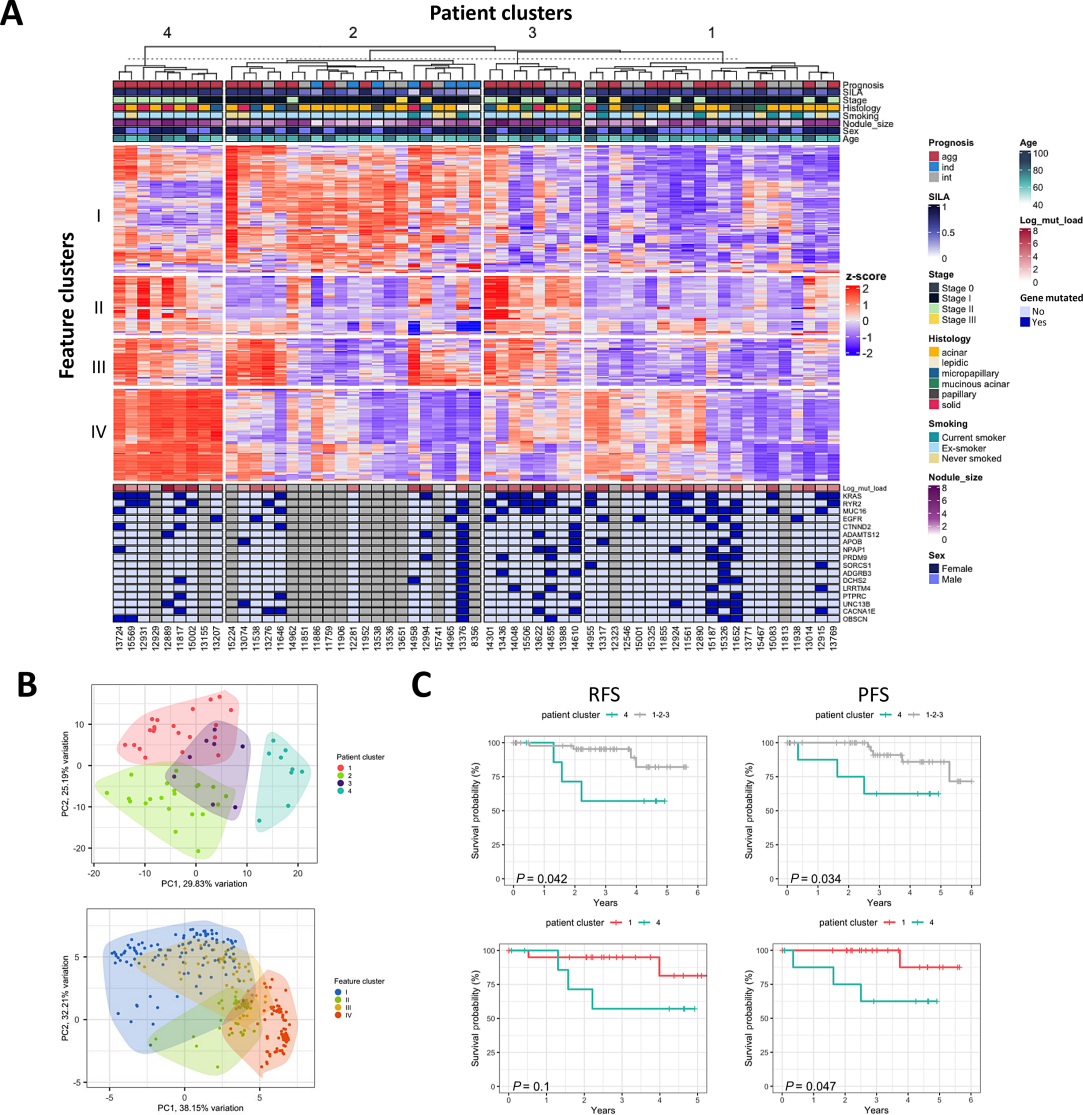
Data integration reveals an association between radiomics features and tumor biology. **A,** Heat map showing the z-score per patient (columns) per feature (rows) split by clusters. Top annotation shows some clinical characteristics and bottom annotation shows mutated genes. **B,** PCA of patients (top) and features (bottom) colored by cluster. **C,** RFS (left) and PFS of patients from cluster 4 versus 1, 2, 3 (top) and 4 versus 1 (bottom).

To this end, we have obtained several features at different biological and clinical levels that are significantly associated with the SILA score and therefore with the predicted level of aggressiveness of the tumors. Using those results as our feature selection strategy, we integrated a total of 301 features from the CyTOF, RNA-seq, and radiomics datasets on 59 patients with complete data across those modalities (Fig. 4; Supplementary Tables S11 and S12). From the RNA-seq dataset, we used the significantly dysregulated pathways from the GSEA (Fig. 3B and C; Supplementary Tables S7–S9) to avoid redundancy. We used the GSVA algorithm to compute individual pathway scores for each patient sample. Features were scaled, centered, and then clustered, resulting in four feature clusters (I–IV; Fig. 4A, see Materials and Methods for details). Feature cluster I (FI) included all CyTOF features that were significantly enriched in indolent tumors (HLA-DR+ subpopulations), and bulk HLA-DR protein expression. It also included the five radiomics features that were negatively correlated with SILA score such as percent glass ground opacity (GGO), root mean square, and surface area to volume ratio (Supplementary Table S10). From the gene expression data, we observed pathways associated with immune response, antigen presentation, cytokine cascades, etc.; pathways associated with tumor initiation and growth signals such as *NOTCH1* and *MYC* but also pathways associated with tumor suppression such as *TP53* and PTEN signaling; and finally, pathways associated with apoptosis, hypoxia, and reactive oxygen species. All these features together suggested a scenario in which the tumors were initiating or attempting growth but opposing signals were fighting back to prevent proliferation and the immune response could either be the cause or the consequence of this process. Feature cluster II (FII) included mostly radiomics features positively correlated with SILA score, a CyTOF subpopulation (ECC5) enriched in aggressive tumors, and the pathways “O-linked glycosylation of mucins” and “KRAS signaling down.” Feature cluster III (FIII) included the radiomics feature “GLCM homogeneity” and then pathways associated with structural components such as collagen degradation and formation, ECM organization, angiogenesis, cell motility, and EMT. GLCM stands for gray-level co-occurrence matrix, also known as the gray-level spatial dependence matrix, and it is a statistical method of examining texture that considers the spatial relationship of pixels (48). The GLCM functions characterize the texture of an image by calculating how often pairs of pixels with specific values and in a specified spatial relationship occur in an image, creating a GLCM, and then extracting statistical measures from this matrix. One of these functions is the homogeneity and it measures the closeness of the distribution of elements in the GLCM to the GLCM diagonal. From our data, we can say that GLCM homogeneity is positively correlated with pathways associated with structural components, and from our pathway analysis we saw that those were upregulated in indolent and aggressive tumors when compared with the intermediate group. One hypothesis is that these groups could be using the structural pathways for different purposes. For example, the aggressive group, as we saw they have an enhanced proliferative profile we could say that these tumors are growing and preparing for metastasis. On the other hand, the indolent tumors which we saw have a more immunogenic profile, we could say that they are using the structural components to facilitate immune cell mobilization and infiltration. Finally, cluster IV (FIV) was composed by pathways associated with cell proliferation, mitosis, DNA replication, and cell cycle. When we performed a PCA on the features clusters and plotted the first two components (*>*70% of variance explained), we observed that FI and FIV showed almost no overlap, whereas FIII mostly overlapped with FI, and FII overlapped mostly with FIV (Fig. 4B, bottom). To better understand those overlapping features, we generated similarity matrix (Supplementary Fig. S13). These results show that there is an almost exclusive expression of either features from FI or FIV, and that some radiomics features from FII behave very similarly to features from FIV. This suggests a potential of using radiomics features to predict the degree of proliferative activity of the tumor. We then clustered the patients to find groups with similar feature characteristics and we found four clusters (1–4; Fig. 4A). Patient cluster 1 (P1) was expressing low levels of most of the feature's clusters, except for a subset of it that were expressing moderate levels of FIV. Patient cluster 2 (P2), was a group of patients with moderate to high levels of FII and low levels of FIV, and a subset of them presented high levels of FIII. Patient cluster 3 (P3) presented moderate levels of FII and FIII and low levels of F1 and FIV. Finally, patient cluster 4 (P4) was characterized for a high level of FIV, moderate levels of FII and FIII, and low FI. When we performed a PCA on the patient clusters and plotted the first two components (*>*55% of variance explained), we observed that clusters P1, P2, and P4 were different from each other, while P3 overlapped with P1 and P2 (Fig. 4B, top). Finally, when we assessed the recurrence-free survival (RFS) and progression-free survival (PFS) of the patient clusters, we found that patients from P4 had the worst prognosis when compared with the other three clusters and when compared with P1 alone. Altogether, these results demonstrated the feasibility of integrating data from different modalities to obtain insights on the tumor biology which can be linked to clinical outcomes.

### In-depth Profiling of the LUAD TME by Single-cell RNA-seq Analysis

**FIGURE 5:**
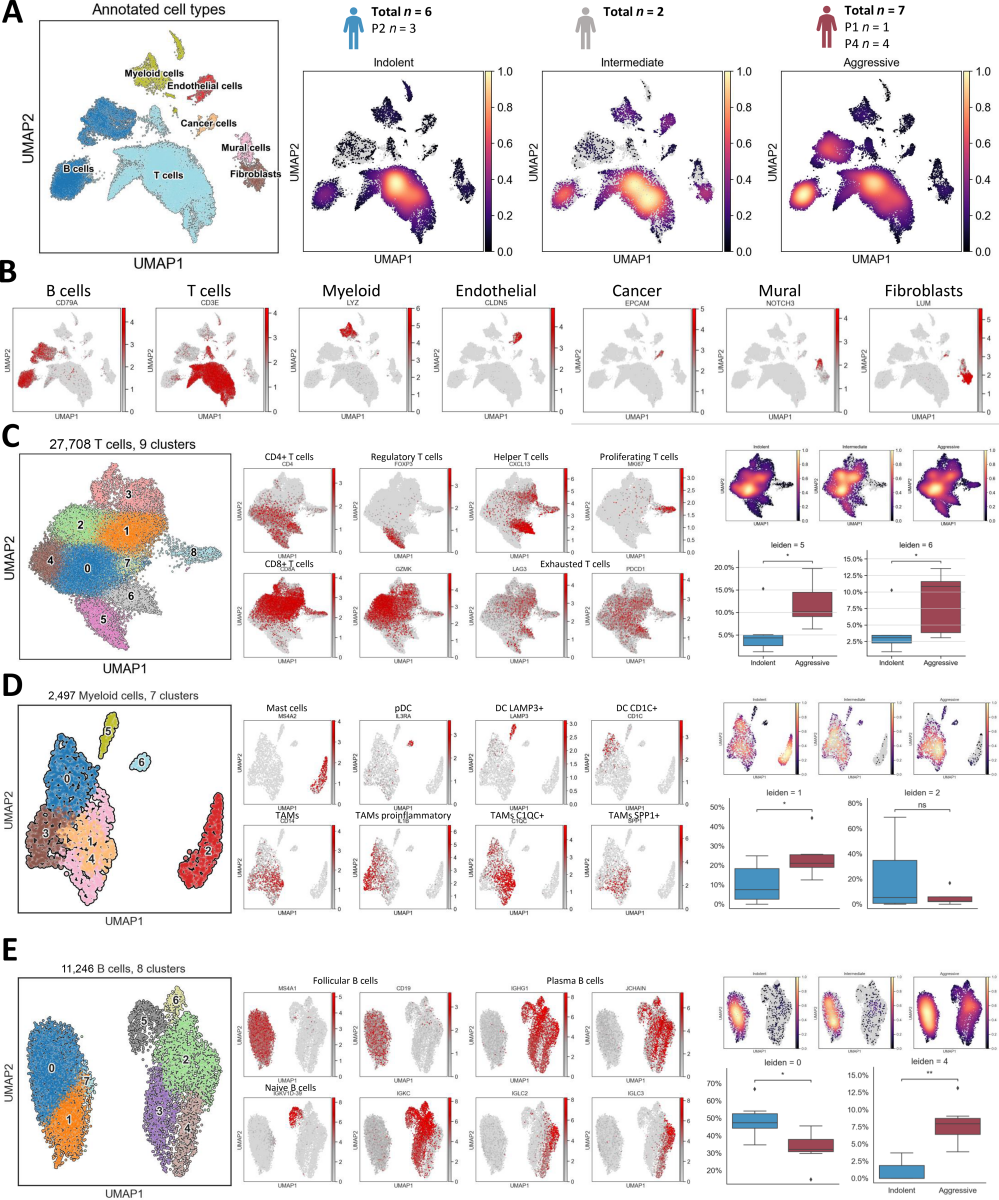
Profiling of LUAD TME by single-cell RNA-seq analysis. **A,** Uniform Manifold Approximation and Projection (UMAP) representation colored by cell type using all cells (left) and by density grouped by risk group (right). **B,** UMAP representation colored by gene expression of top lineage gene markers for each main cell type. Reclustering analysis for T cells (**C**), myeloid cells (**D**), and B cells (**E**). UMAP representation colored by cluster, followed by UMAP representation colored by gene expression of some subset representative markers. On the far right, we have UMAP by density grouped by risk group (top) and differential abundance analysis (bottom). *Y* axis corresponds to the fraction of cells per patient sample. ns, *P* > 0.05; *, *P* < 0.05; **, *P* < 0.001.

To better understand the microenvironment of tumors with different predicted behavior, we performed single-cell RNA-seq of 15 tumors (indolent *n* = 6 of which 3 were P2, intermediate *n* = 2, aggressive *n* = 7 of which 1 was P1 and 4 P4; Fig. 5). After quality filtering (see Materials and Methods), we obtained 44,867 cells. Out of these, 14,795 cells (%33) came from indolent tumors, 7,107 cells (%16) from intermediate tumors, and 22,974 (%51) from aggressive tumors. After gene normalization and filtering, we applied PCA to 1,871 highly variable genes, and performed a graph-based clustering (42) to classify the cells into groups of similar gene expression. We annotated those clusters and identified seven major cell types: B cells, T cells, myeloid cells, endothelial cells, cancer cells, mural cells, and fibroblasts (Fig. 5A and B; Supplementary Fig. S14). Aggressive tumors were significantly enriched in B cells, while indolent tumors showed a significantly higher proportion of T cells (Supplementary Fig. S14C), and we see a similar pattern for patients from P4 and P2, respectively (Supplementary Fig. S14B). We then performed an additional clustering step to find subclusters within each of these main cell types (Fig. 5C and E; Supplementary Fig. S15–S21). In the T-cell group, we obtained nine clusters (Fig. 5C; Supplementary Fig. S15). Clusters 0, 4, 5, 6, and 7 were identified as *CD4*^+^ T cells and clusters 1, 2, and 3 were identified as *CD8*^+^ T cells. Clusters 5 and 6 were significantly enriched in aggressive tumors compared with indolent. Cluster 5 showed high *FOXP3* expression which is characteristic of regulatory T cells (Treg), whereas cluster 6 showed high expression of *CXCL13*, a chemokine expressed by Th cells. Numerous *CD8*^+^ T cells also expressed *GZMA*, *GZMB*, *GZMK,* and *GNLY*, which encode the cytotoxic molecules granzymes A, B, and K and granulosyn, respectively. In addition to granzymes and other cytotoxic molecules, cluster 3 also expressed *FCGR3A*, a gene that encodes CD16, which presumably indicates that these are natural killer T cells (Supplementary Fig. S15B). Cluster 8 corresponded to proliferating T cells, both *CD8*^+^ and *CD4*^+^. A fair number of cells, particularly those in cluster 6 were expressing *LAG3* and *PDCD1*, markers of T-cell exhaustion. When we look at the samples classified by the data integration clusters from Fig. 4, patients from P4 and P2 followed similar patterns as aggressive and indolent, respectively, while the patient from P1 behaved like the indolent group but with less concentration of cytotoxic T cells (Supplementary Fig. S15A). In the myeloid cell compartment, we found seven clusters, from which clusters 1, 3, and 4 were tumor-associated macrophages (TAM) expressing genes such as *HLA-DRB1* and *CD14* (Fig. 5D; Supplementary Fig. S16). Cluster 3 was enriched in proinflammatory TAM markers such as *IL1B*, while clusters 4 and 1 expressed *C1QC* and *SPP1* genes. Clusters 0, 5, and 6 were dendritic cells (DC), with 0 being *CDC1*^+^ DCs, 5 being *LAMP3*+ DCs, and 6 being plasmacytoid DCs expressing *IL3A*. Finally, cells from cluster 2 were identified as mast cells for their unique expression of *MS4A2*. Aggressive tumors as well as P4 tumors were enriched in cluster 1, while the mast cell subset (cluster 2) was dominated by one particular indolent tumor (11522; Supplementary Fig. S16A, S16C, and S16D). In the B-cell compartment we found eight clusters, from which clusters 0, 1, and 7 corresponded to follicular B cells, given their expression of *MS4A1* and *CD19* and HLA-DR–related genes (Fig. 5E; Supplementary Fig. S17B). Cluster 5 was identified as naïve B cells, and clusters 2, 3, 4, and 6 were plasma B cells. Indolent tumors, but no P2 tumors, were enriched in cluster 0, and aggressive tumors were enriched in cluster 4. Tumors from P2 had little to no fraction of B cells in general, while tumors from P1 and P4 behaved similarly to each other and were similar to aggressive tumors (Supplementary Fig. S17A).

Mural cells are composed by six clusters, from which clusters 0, 1, 2, 4, and 5 are characterized by the expression of some collagen genes, *NOTCH3*, *ACTA2*, *PDGFRB* which are commonly expressed in smooth muscle cells (SMC), and cluster 3 is characterized by the expression of *KLF4* and *MGP*, genes associated with mesenchymal cells and regulation of SMC. Indolent and P2 are slightly enriched in cluster 3 cells while aggressive tumors appear to be enriched in cluster 0 cells (Supplementary Fig. S18). In the fibroblasts compartment, we found seven clusters, from which both indolent and aggressive tumors were enriched in clusters 1 and 3, which were characterized for the expression of various collagen genes including *COL1A1* and *COL1A2*, and intermediate tumors were enriched in cluster 2, characterized by the expression of some *MFAP4*, *A2M*, *LIMCH1*, among other genes involved in cell adhesion, protease inhibition and cell migration (Supplementary Fig. S19). In terms of the data integration patient groups, P2 and P4 were also enriched in clusters 1 and 3. In the endothelial compartment, we found seven clusters; however, most of these cells come from patients 14428 (intermediate) and 13634 (indolent; Supplementary Fig. S20). Finally, in the cancer cell compartment, few indolent tumor cells were present, intermediate tumors were enriched in cluster 1, and aggressive tumors were enriched in cluster (Supplementary Fig. S21). Cells from cluster 1 were characterized for the expression of some HLA-DR–related genes, as well as lung-specific markers *SFTPB* and *MUC1*. Cells from cluster 0 expressed the collagen III gene *COL3A1* and *MIF*, a gene that encodes the macrophages migration inhibitory factor.

To recapitulate some of the main findings of this section, indolent tumors show higher percentage of T cells compared with aggressive tumors, but aggressive tumors are significantly enriched in Tregs and Th cells. Aggressive tumors show a higher percentage of B cells compared with indolent tumors, which can be explained by a lack of plasma B cells in the latter. Aggressive tumors also show a higher percentage of *CD14*^+^/*C1QC*+/*SPP1*+/*IL1B*− TAMs. Indolent tumors also present an enrichment in mesenchymal mural cells, while aggressive tumors seem to be enriched in SMC-like cells, which correlates well with a more solid tumor component. The interesting finding from the RNA-seq dataset in which both indolent and aggressive tumors appear to share an upregulated signature for structural cellular pathways could be explained by looking at the fibroblasts compartment, in which tumors from both groups have an enrichment in fibroblasts with high expression of several collagen genes. In summary, these results give us a deeper understanding of the cellular subsets in LUAD and their transcriptomic profiles which help us to better understand the biological differences between indolent and aggressive tumors.

## Discussion

Understanding the biology of LUADs in the context of tumor behavior is crucial to improving the current clinical standards of diagnosis and treatment, particularly in early stages of the disease. In this study, we presented a comprehensive set of patients with early-stage LUAD risk-stratified into predicted indolent, intermediate or aggressive behavior groups based on radiomics, with data collected across different biological layers. First, we used our previously validated CyTOF panel (20) to assess the difference between indolent and aggressive tumors at the proteomic level (2). We found that indolent tumors were significantly enriched in a subset of cancer cells and a subset of fibroblast/mesenchymal cells characterized by high HLA-DR protein expression, compared with aggressive tumors, and that these subsets were positively correlated with CD8^+^ T cells, CD4^+^ T cells, and myeloid cell abundance. This is an example of what is known in Probability as the “Simpson's paradox” (49), a phenomenon in which a trend appears in several different groups of data but disappears or reverses when these groups are combined. Although those gross immune cell types are not significantly enriched in any of our groups, when the cancer and fibroblasts/mesenchymal cells compartments were broken into subpopulations, a correlation between some of those and the immune cell types emerged, and those cancer subpopulations were in fact significantly enriched in indolent or aggressive groups. HLA-DR bulk protein expression was also significantly higher in indolent versus aggressive tumors. We previously showed that HLA-DR expression was enriched in indolent tumors and that it was correlated with an increased abundance of T cells (20). In the current study, we were able to confirm those CyTOF results in a bigger cohort and the other data modalities also suggested an increased immune response in indolent tumors compared with aggressive. While MHC-II expression is usually restricted to antigen-presenting cells (APC), it has been shown that its expression can also be induced in non-APCs in response to an inflammatory microenvironment and there is evidence of MHC-II molecule expression in cancer cells associated with good prognosis in various cancer types such as melanoma, breast cancer, and esophageal cancer (50–54). In a recent study (55), the authors assessed the effect of cancer cell–specific MHC-II expression in LUAD on T-cell recruitment to tumors and response to anti-PD-1 therapy in murine models. They found that loss of *CIITA*, a master regulator of the MHC-II pathway, decreased MHC-II expression in cancer cells and made the cells resistant to anti-PD1. This effect was associated with reduced levels of Th1 cytokines, reduced T-cell infiltration and macrophage recruitment, and increased B-cell abundance. The opposite occurred with enforced expression of *CIITA*. They validated these results in surgically removed human LUADs, showing that MHC-II expression improved survival and positively correlated with T-cell expression. These results align well with our findings in the CyTOF dataset, which mainly revolve around the high correlation of EpCAM and HLA-DR protein expression in indolent tumors and highlight the potential of MHC-II expression in cancer cells as an independent biomarker of sensitivity to checkpoint inhibitors. In our single-cell RNA-seq data, we found that indolent tumors were enriched in T cells, but aggressive tumors were enriched in Tregs and Th cells specifically (5). Also, aggressive tumors were enriched in B cells and indolent tumors mostly lacked plasma B cells. The influence of plasma B cells in non–small cell lung cancer has been mostly studied in the context of immunotherapies or adjuvant chemotherapies, in which cases it has been associated with improved prognosis (56, 57). However, is important to note that most of these tumors are late stage or metastatic. We then investigated the difference in gene expression between tumors of different predicted behavior (3). When comparing indolent versus aggressive, the serotonin transporter *SLC6A4* was the top downregulated gene. It has been reported to be overexpressed in normal lung compared with LUAD and its deregulation has been associated with tobacco consumption (58, 59). *KIF1A* and *HMGA2* were some of the top upregulated genes in aggressive tumors, the first one has been associated with drug resistance in breast cancer (60, 61) and the latter was reported to be associated with reduced overall survival in patients with LUAD, positively regulating lung cancer proliferation, progression, and metastasis (62, 63). In the GSEA, when comparing aggressive versus indolent or intermediate, pathways associated with proliferation and cell cycle were upregulated, and when comparing indolent versus aggressive or intermediate, pathways related with immune response were upregulated. It is intriguing, though, given the strong proliferative gene expression profile in aggressive tumors that we only see a mild and nonsignificant enrichment of Ki67 protein expression in these tumors. An additional protein expression assay with a different method such as IHC could provide a clearer picture; however, we are limited to the data presented here. Although we found that the Hallmark pathway Allograft rejection (37), a gene set that includes MHC-I– and MHC-II–related genes as well as granzymes and cytokines such as IFNG, was upregulated in indolent tumors, pathways related with antigen presentation were not, suggesting that the high HLA-DR protein expression we saw in indolent tumors might be a consequence of an inflammatory microenvironment rather than the cause of inflammation by antigen presentation. An unexpected finding appeared when we compared either aggressive or indolent versus intermediate. Patients from both extremes shared upregulation of pathways related to structural functions such as ECM organization, collagen formation and degradation, EMT, etc. These patients also presented an increased inferred activity of the *HIF-1* alpha TF, which is a master regulator of cellular and systemic homeostatic response to hypoxia (64, 65). One possible explanation is the dual effect of some of these actors. For example, *HIF-1* alpha may promote both tumorigenesis and apoptosis under different circumstances (66). In that study, the authors claim that most of the conflicting data can be explained by the different cutoffs used to define high *HIF-1* alpha expression. They analyzed the expression of *HIF-1* alpha in NSCLC by IHC, defining as low cutoff the median staining (>5%) and as high cutoff >60%, and found that when using the latter an association with poor prognosis was significant. In a recent study of ours (67) using the same LUAD patient samples, we described in a previous study (20), we found, by multiplex immunofluorescence, that indolent and aggressive tumors did not show significant difference in neither the amount of collagen fibers nor the average fiber length. However, when we performed spatial analysis, we found that tumor cells from the indolent group were colocalized with an increased number of immune cells. In addition, tumor cells from aggressive LUADs were colocalized with lower number of collagen fibers and these fibers generally had smaller length, which may indicate involvement of these cells in the processes of collagen degradation and ECM remodeling. It is known that increased collagen deposition also increases the stiffness of the tumor, and this has been associated with poor prognosis in several cancer types (68). Some *in vitro* studies show that T cells migrate slower through collagen gels of high density compared with low density (69, 70). Other *in vitro* studies have also demonstrated that T cells preferentially migrate along the collagen fibers, indicating that the collagen orientation could control the migration of T cells (71). The overexpression of these signatures in our cohort could also suggest that both tumor types have the potential for metastasis, but indolent tumors have other tools to counteract these while aggressive tumors have tools to support them. In addition, when we investigated the fibroblasts compartment in our single-cell RNA-seq data (Supplementary Fig. S19), we see similarities between indolent and aggressive tumors; however, in the mural cells compartment aggressive tumors appear to have higher density of smooth muscle–like cells which show high collagen expression compared with other cells in this subset (Supplementary Fig. S18). We also see a higher number of Tregs and Th cells in aggressive tumors, which has been associated with a stiffer microenvironment (72). In that study, collagen led to an increase in the CD4:CD8 ratio among the infiltrating T cells and the CD4^+^ T cells were skewed toward a Th2 phenotype. We then integrated biological and radiomics features that were significantly associated with tumor behavior (4). We found four main feature signatures: (i) immune response, growth initiation signals, and tumor suppression; (ii) radiomics features positively correlated with SILA; (iii) ECM organization and other structural components; (iv) proliferation and cell cycle. (i) and (iv) were strongly negatively correlated, and some features from (ii) such as percentage of solid component were positively correlated with (iv), while percent of Ground Glass Opacity (GGO) was positively correlated with (i). Multiple radiomics studies and tools have focused on prediction of invasiveness, and association of solid or GGO component with outcome. Our results are in agreement with the literature in that tumors with increased GGO percent show improved prognosis whereas tumors with higher solid percentage are associated with poor survival (13, 15, 16, 73). However, there is no study in LUAD at the moment that has demonstrated correlation between radiomics features and specific and detailed biological signatures such as cell cycle, proliferation, DNA replication, mitosis, immune response, etc. We demonstrated a strong positive correlation between features associated with solid components and proliferation signatures, and these were also strongly but negatively correlated with immune response (Fig. 4). Similarly, GGO and other radiomics features negatively correlated with SILA showed an opposite relationship. This is a unique and unprecedented finding that connects a tool widely use in the clinic with biological insights of the tumor.

Our results show a potential novel bridge between tumor biology and the developing field of radiomics. However, our work also has its limitations. In the clinic, there are fewer patients that come with indolent tumors than aggressive ones, therefore our cohort has a reduced number of these samples which limits the study of intrapatient heterogeneity in this subset and introduces some degree of bias as we have an overrepresentation of aggressive tumors. In the same line, aggressive tumors are, for the most part, bigger than indolent tumors, which inherently influences the total number of cells and thus our ability to capture intracellular heterogeneity. These tissues are also less affected by cell loss during tissue processing. As for clinical limitations, the approach to define the aggressiveness or indolence of LUAD is still at the discretion of the researcher as there is no gold standard. The behavior of LUADs is confounded by the heterogeneous treatments patients undergo and we do not know the true natural history of early LUAD, as prospective studies to simply observe the natural history of the tumor without intervention would be unethical. In this study, all patients had resection of their primary lung nodule and an accompanying CT scan of that nodule obtained a few weeks or days before surgery. We decided to use SILA, a CT-based tool that predicts the degree of histologic tissue invasion and patient survival specifically design for LUAD. We acknowledge that this, as any other predictive tool, is not flawless but it has been thoroughly validated to accurately correlate with histopathologic assessment (14). Even though the use of clinical prognosis or outcomes is preferred for group classification in these types of studies, in our cohort some of the patients have less than 5 years of follow-up data, which is the minimum typically used to correlate with survival. As for experimental limitations, each dataset that we presented in this study has its own limitations and its own biases. For instance, the CyTOF dataset is limited to a fixed number of proteins compared with single-cell RNA-seq in which thousands of transcripts can be analyzed. Yet, the latter is affected by sparsity of reads and the cost limits the number of samples and number of cells to be sequenced. In addition, both datasets require the tumor to be processed to obtain single cells, introducing an additional component of perturbation to the system, and incidentally selecting for some cell types. The RNA-seq and WES technologies are much more affordable, facilitating sequencing of more samples but the results can only be interpreted as bulk tissue expression, not by single-cell analysis. Despite these limitations, the strength of this study is to have all those datasets together to fill in the missing pieces. Although we present unique findings in each dataset, we were also able to find a common thread and results that complement each other. Finally, we would like to acknowledge the absence of a validation cohort. The main reason being that a cohort with the same characteristics and variety of datasets does not exist publicly and producing one involves substantial effort and resources. The Cancer Genome Atlas datasets would be the closest; however, their CT scans are decades old with much lower resolution than the current ones, making them incompatible with our data. It is important to note that the main aim of our study is not to validate a new algorithm or tool, but rather to use multi-omics analysis to highlight the links between radiomics and tumor biology as an exploratory, proof-of-concept, and data generation study which will feed hypothesis-driven studies.

In conclusion, we presented a unique and comprehensive collection of datasets in LUAD from which we were able to elucidate previously unknown insights in the biology of the tumors related to their predicted behavior, and data integration provided an evident and unprecedented link between tumor biology and structural radiomics. We also showed the important role of the TME, both in the immune compartment and the stromal compartment, in defining the indolence or aggressiveness of the tumors. Finally, experimental and mechanistic validations are needed to further understand these relationships. This is a rich data collection with huge potential that could be further explored in the future to answer multiple other research questions regarding LUAD. We believe that this work contributes to the knowledge and characterization of LUAD tumor biology in relation with its indolence or aggressiveness and further research can potentially integrate this evidence into the clinical settings to improve current management of early LUADs.

## Supplementary Material

Supplementary Tables S1-S12 Supplementary tables

Supplementary Figures S1-S21 Supplementary figures
