# Supplementary material for: Integrated Multi-omics Analysis of Early Lung Adenocarcinoma Links Tumor Biological Features with Predicted Indolence or Aggressiveness
Source: Cancer Res Commun. 2023 Jul 26;3(7):1350–65. doi: 10.1158/2767-9764.CRC-22-0373 (PMC10370362; doi:10.1158/2767-9764.CRC-22-0373)
Supplement: Supplementary Figures S1-S21 — Supplementary figures [file crc-22-0373-s02.pdf]

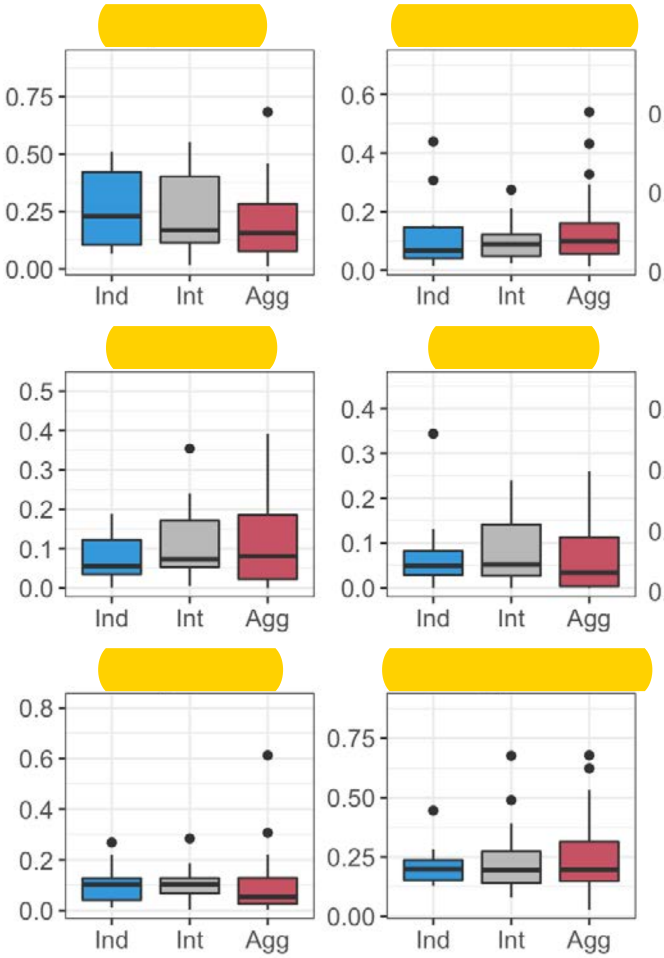

Figure S1. Differential abundance analysis. Y axis corresponds to the fraction of cells per patient sample. P value >0.05 for all comparisons.

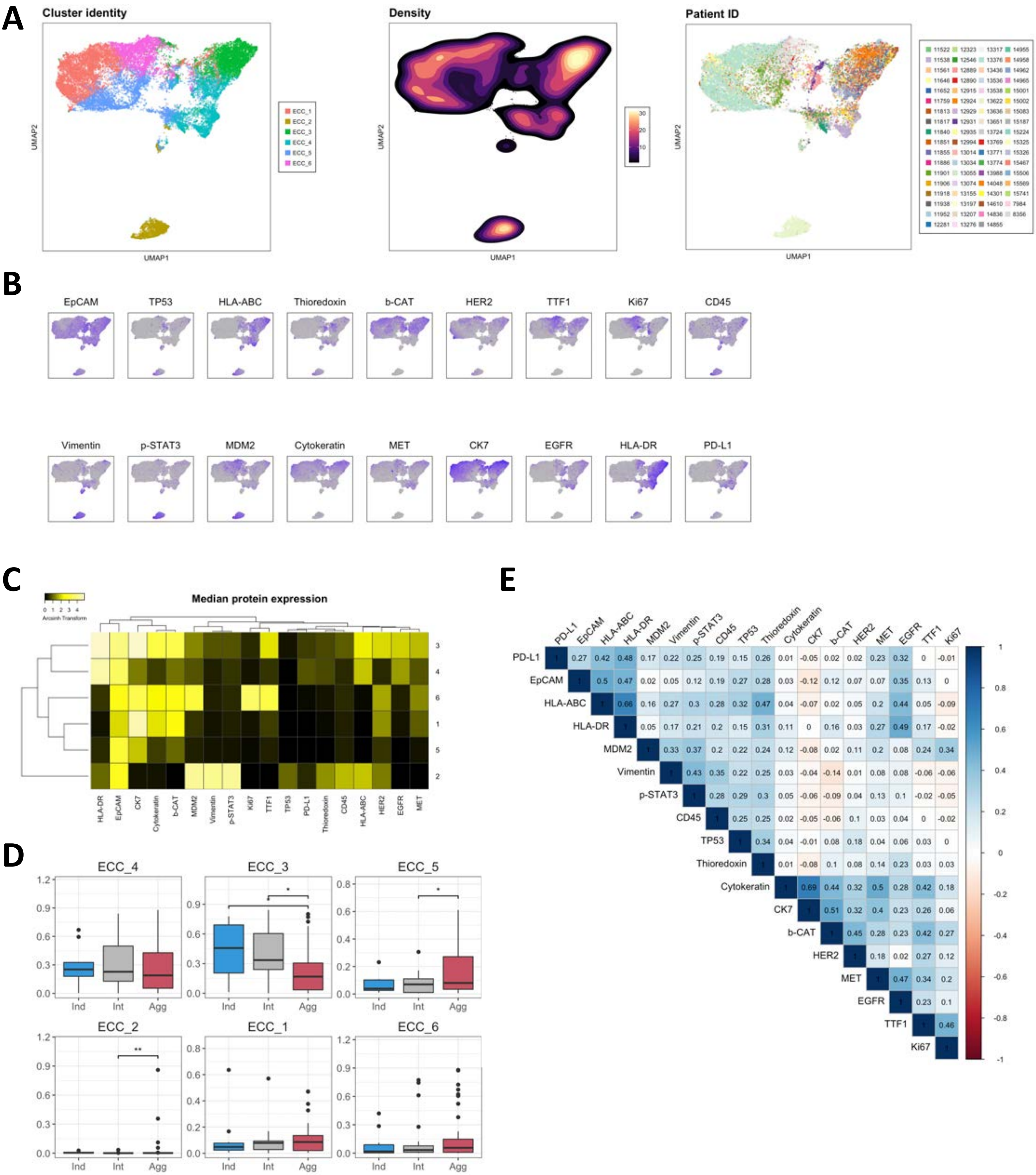

Figure S2. Epithelial cancer cells cluster analysis. (A) UMAP representation of the clusters colored by cluster identity, density, and patient ID. (B) UMAP representation of the clusters colored by protein expression intensity. These are the features used for both clustering and UMAP visualization. (C) Heatmap of median protein expression per protein marker per cluster. (D) Differential abundance analysis. Y axis corresponds to the fraction of cells per patient sample. No star=pvalue>0.05, *=pvalue<0.05, **=pvalue<0.001. (E) Protein-protein Spearman correlation analysis. Only significant correlations (p value >0.05) are colored.

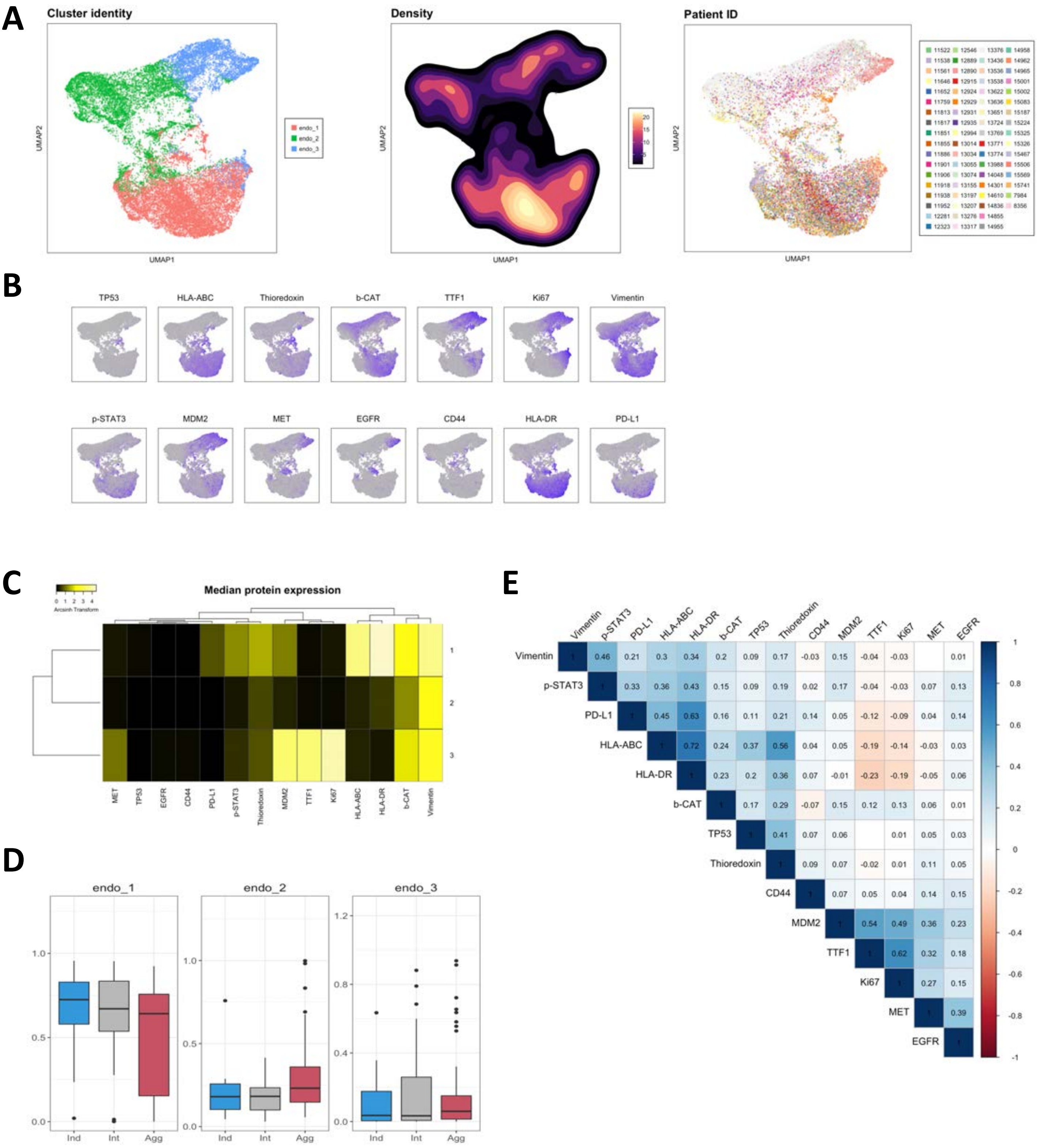

Figure S3. Endothelial cells cluster analysis. (A) UMAP representation of the clusters colored by cluster identity, density, and patient ID. (B) UMAP representation of the clusters colored by protein expression intensity. These are the features used for both clustering and UMAP visualization. (C) Heatmap of median protein expression per protein marker per cluster. (D) Differential abundance analysis. Y axis corresponds to the fraction of cells per patient sample. No star=pvalue>0.05, *=pvalue<0.05, **=pvalue<0.001. (E) Protein-protein Spearman correlation analysis. Only significant correlations (p value >0.05) are colored.

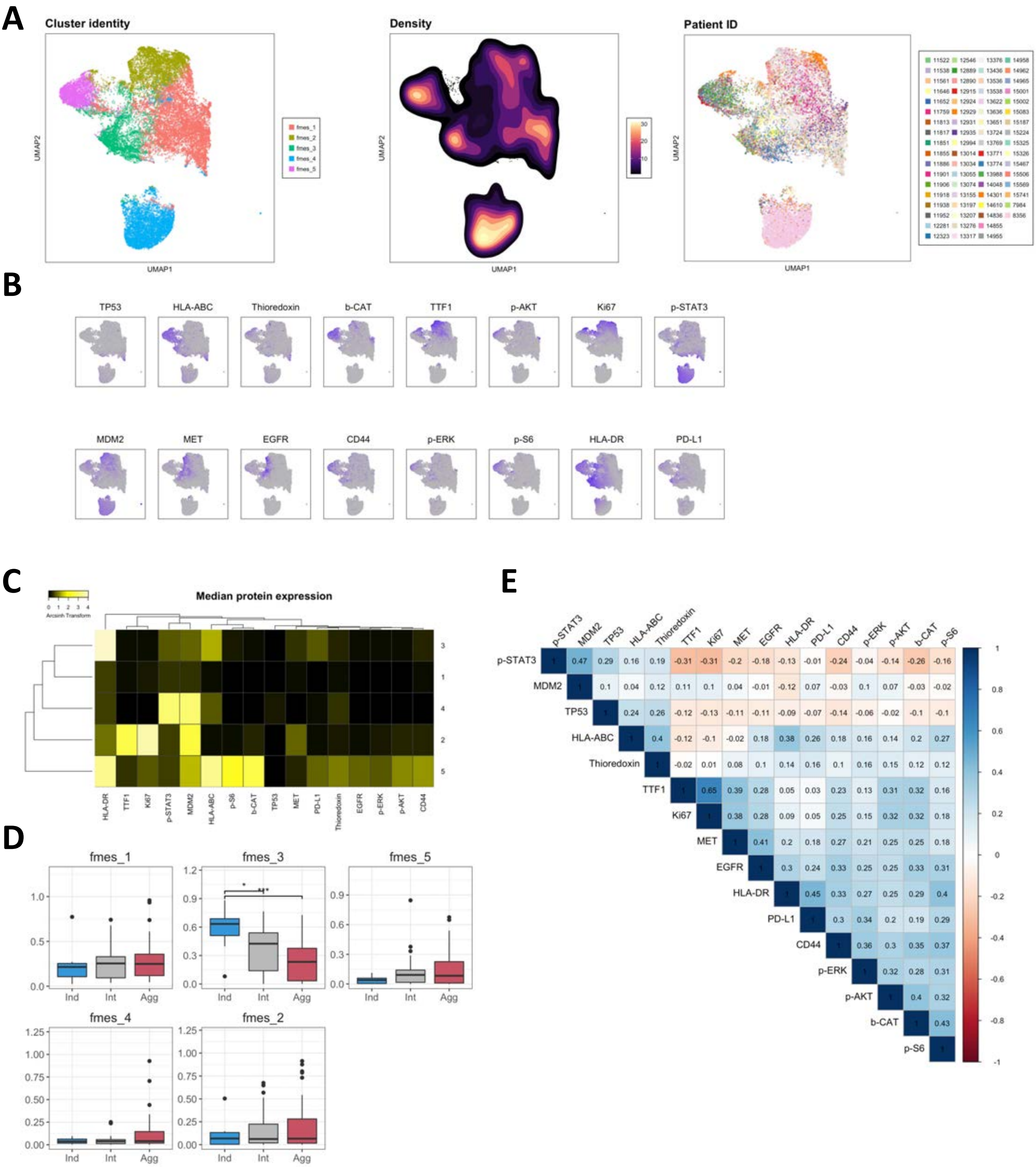

Figure S4. Fibroblasts/Mesenchymal cells cluster analysis. (A) UMAP representation of the clusters colored by cluster identity, density, and patient ID. (B) UMAP representation of the clusters colored by protein expression intensity. These are the features used for both clustering and UMAP visualization. (C) Heatmap of median protein expression per protein marker per cluster. (D) Differential abundance analysis. Y axis corresponds to the fraction of cells per patient sample. No star=pvalue>0.05, *=pvalue<0.05, **=pvalue<0.001. (E) Protein-protein Spearman correlation analysis. Only significant correlations (p value >0.05) are colored.

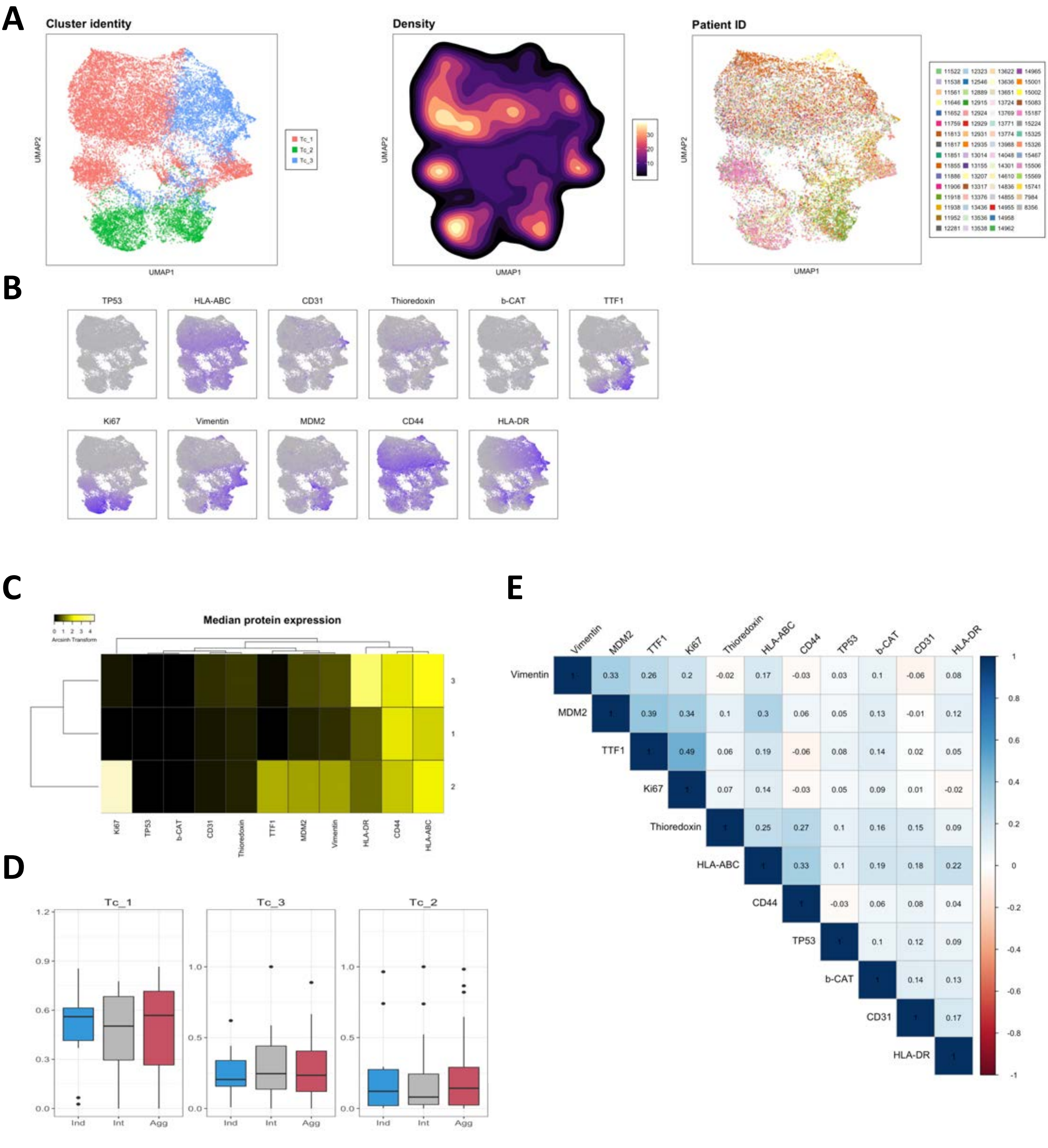

Figure S5. CD8+ T cells cluster analysis. (A) UMAP representation of the clusters colored by cluster identity, density, and patient ID. (B) UMAP representation of the clusters colored by protein expression intensity. These are the features used for both clustering and UMAP visualization. (C) Heatmap of median protein expression per protein marker per cluster. (D) Differential abundance analysis. Y axis corresponds to the fraction of cells per patient sample. No star=pvalue>0.05, *=pvalue<0.05, **=pvalue<0.001. (E) Protein-protein Spearman correlation analysis. Only significant correlations (p value >0.05) are colored.

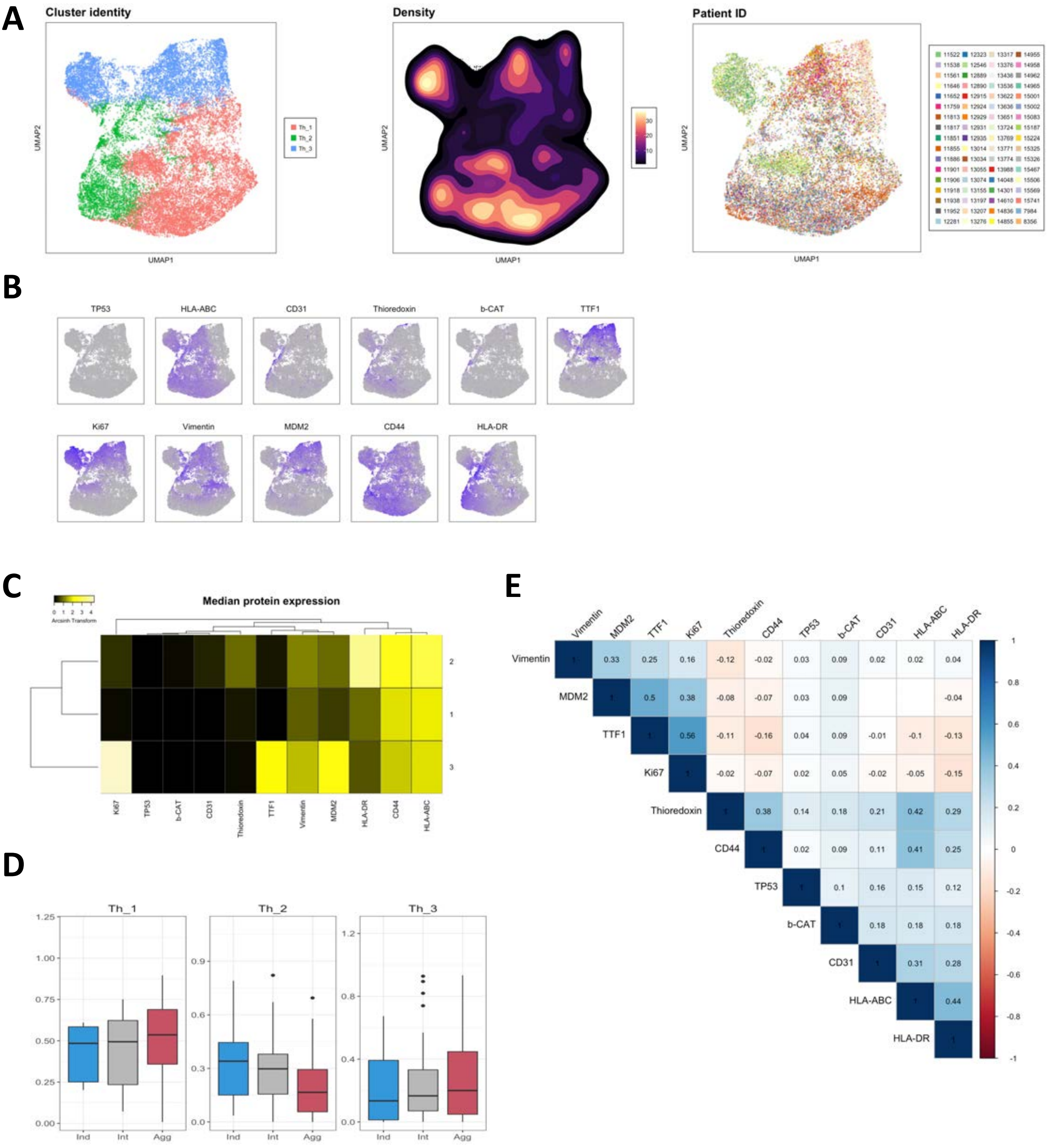

Figure S6. CD4+ T cells cluster analysis. (A) UMAP representation of the clusters colored by cluster identity, density, and patient ID. (B) UMAP representation of the clusters colored by protein expression intensity. These are the features used for both clustering and UMAP visualization. (C) Heatmap of median protein expression per protein marker per cluster. (D) Differential abundance analysis. Y axis corresponds to the fraction of cells per patient sample. No star=pvalue>0.05, *=pvalue<0.05, **=pvalue<0.001. (E) Protein-protein Spearman correlation analysis. Only significant correlations (p value >0.05) are colored.

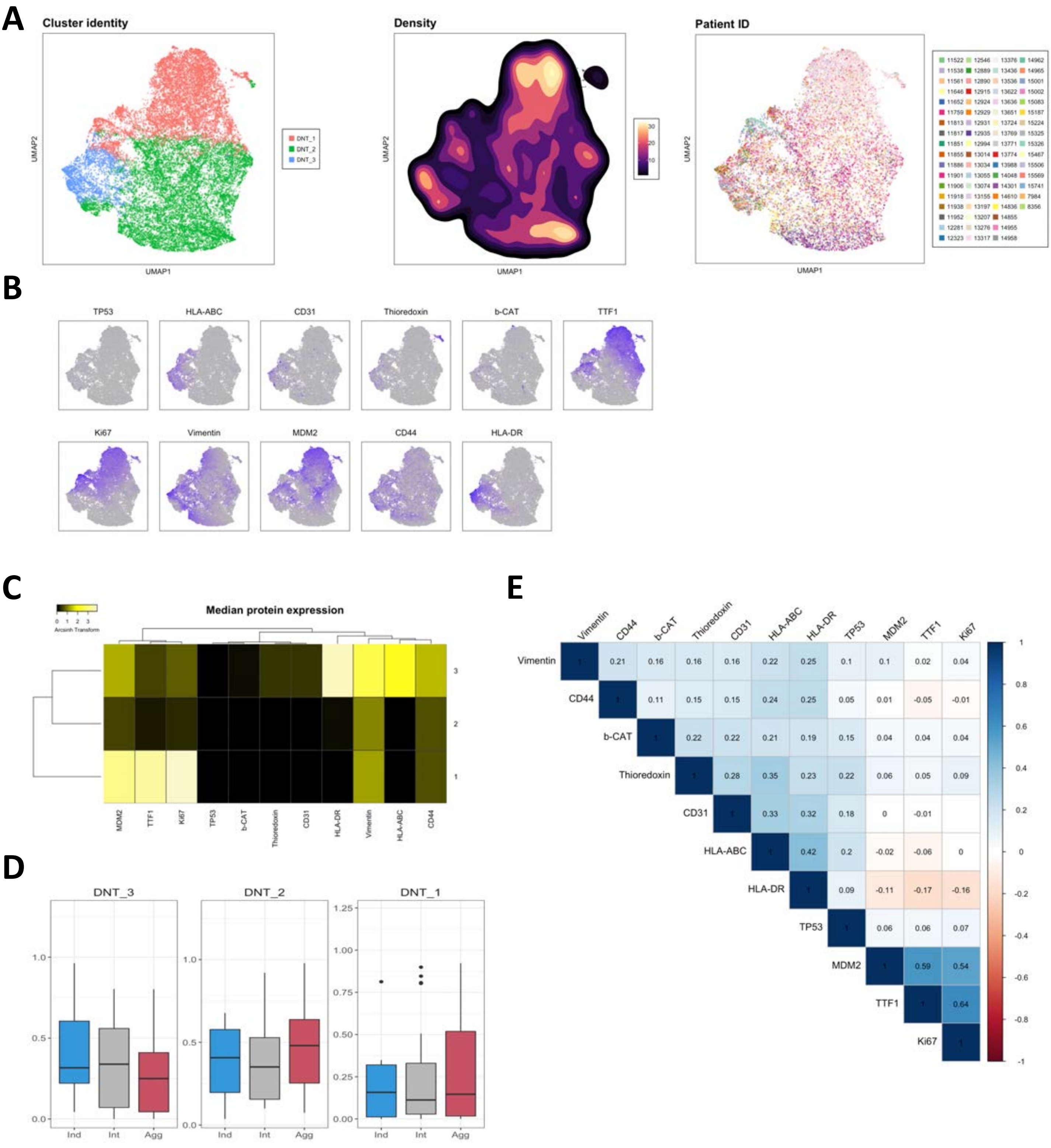

Figure S7. CD8-/CD4- T cells cluster analysis. (A) UMAP representation of the clusters colored by cluster identity, density, and patient ID. (B) UMAP representation of the clusters colored by protein expression intensity. These are the features used for both clustering and UMAP visualization. (C) Heatmap of median protein expression per protein marker per cluster. (D) Differential abundance analysis. Y axis corresponds to the fraction of cells per patient sample. No star=pvalue>0.05, *=pvalue<0.05, **=pvalue<0.001. (E) Protein-protein Spearman correlation analysis. Only significant correlations (p value >0.05) are colored.

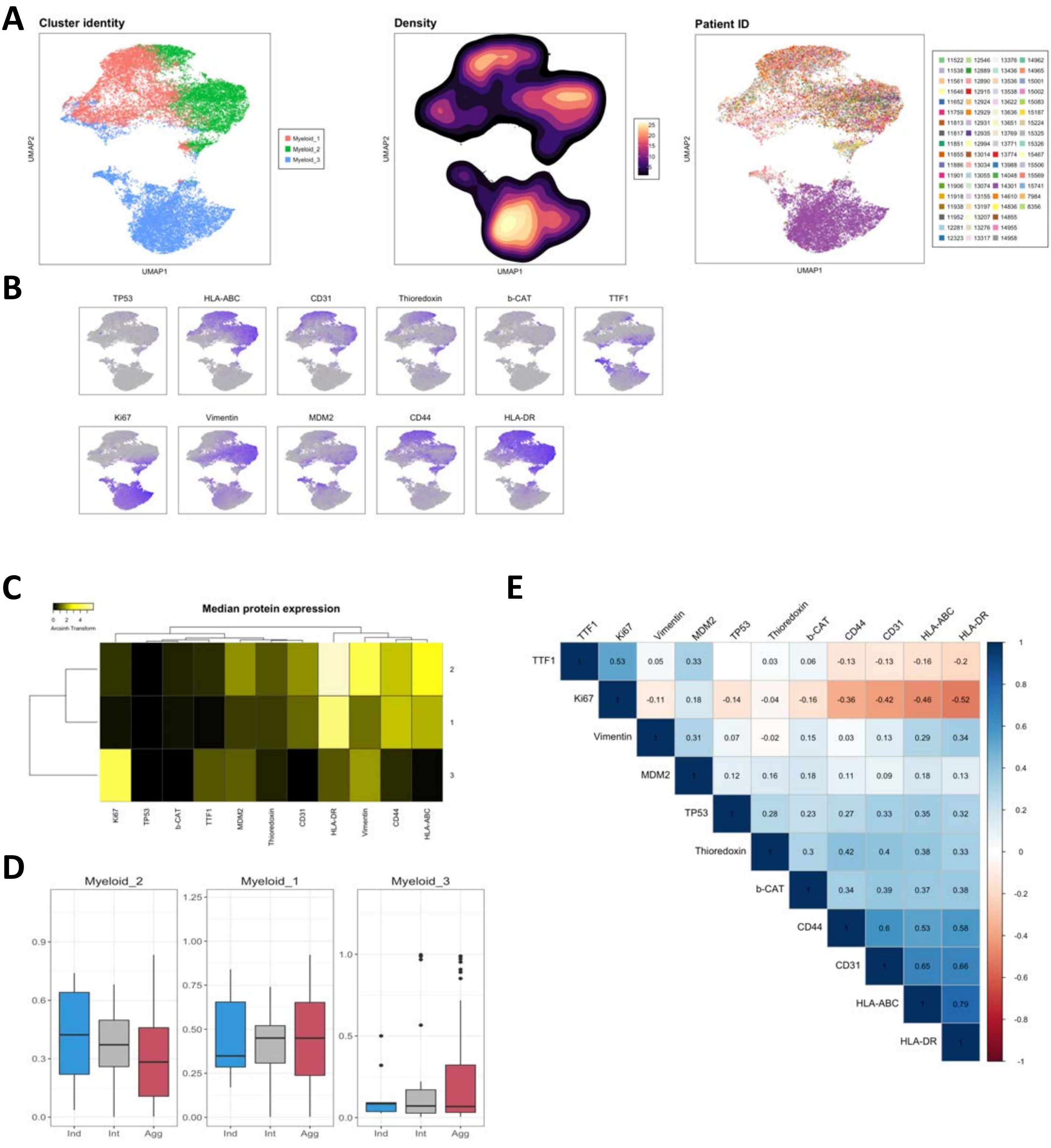

Figure S8. Myeloid cells cluster analysis. (A) UMAP representation of the clusters colored by cluster identity, density, and patient ID. (B) UMAP representation of the clusters colored by protein expression intensity. These are the features used for both clustering and UMAP visualization. (C) Heatmap of median protein expression per protein marker per cluster. (D) Differential abundance analysis. Y axis corresponds to the fraction of cells per patient sample. No star=pvalue>0.05, *=pvalue<0.05, **=pvalue<0.001. (E) Protein-protein Spearman correlation analysis. Only significant correlations (p value >0.05) are colored.

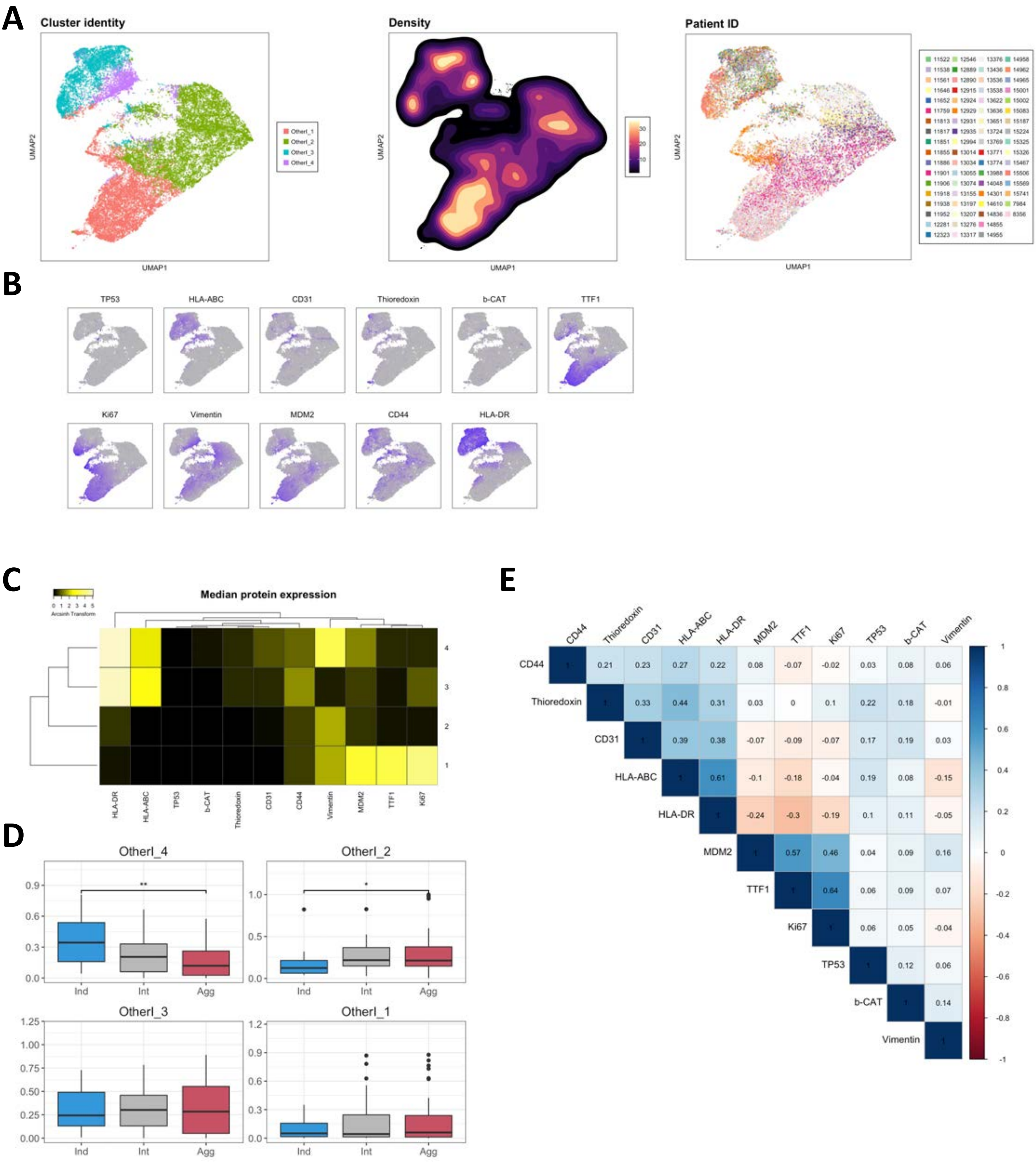

Figure S9. Other immune cells cluster analysis. (A) UMAP representation of the clusters colored by cluster identity, density, and patient ID. (B) UMAP representation of the clusters colored by protein expression intensity. These are the features used for both clustering and UMAP visualization. (C) Heatmap of median protein expression per protein marker per cluster. (D) Differential abundance analysis. Y axis corresponds to the fraction of cells per patient sample. No star=pvalue>0.05, *=pvalue<0.05, **=pvalue<0.001. (E) Protein-protein Spearman correlation analysis. Only significant correlations (p value >0.05) are colored.

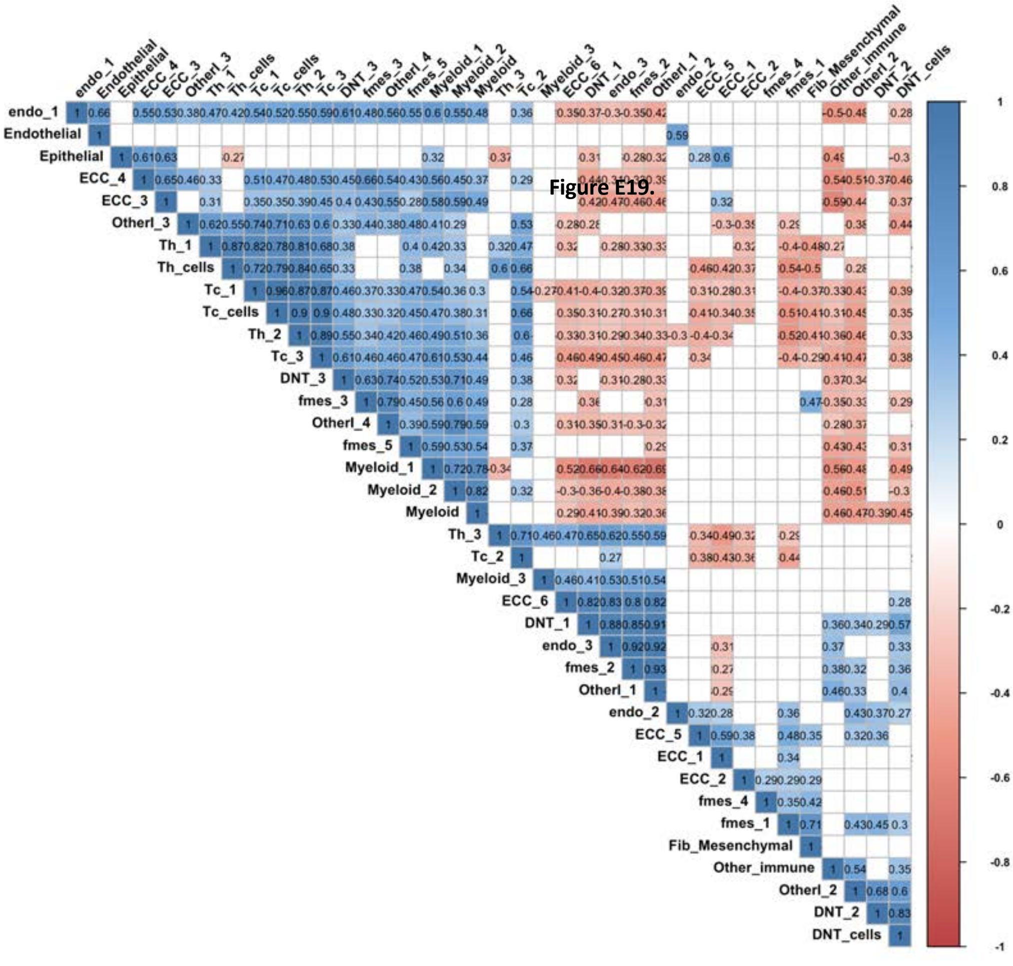

Figure S10. Spearman correlation of fraction per patient sample of main cell types and cell types clusters. Only significant correlations (p value >0.05) are colored.

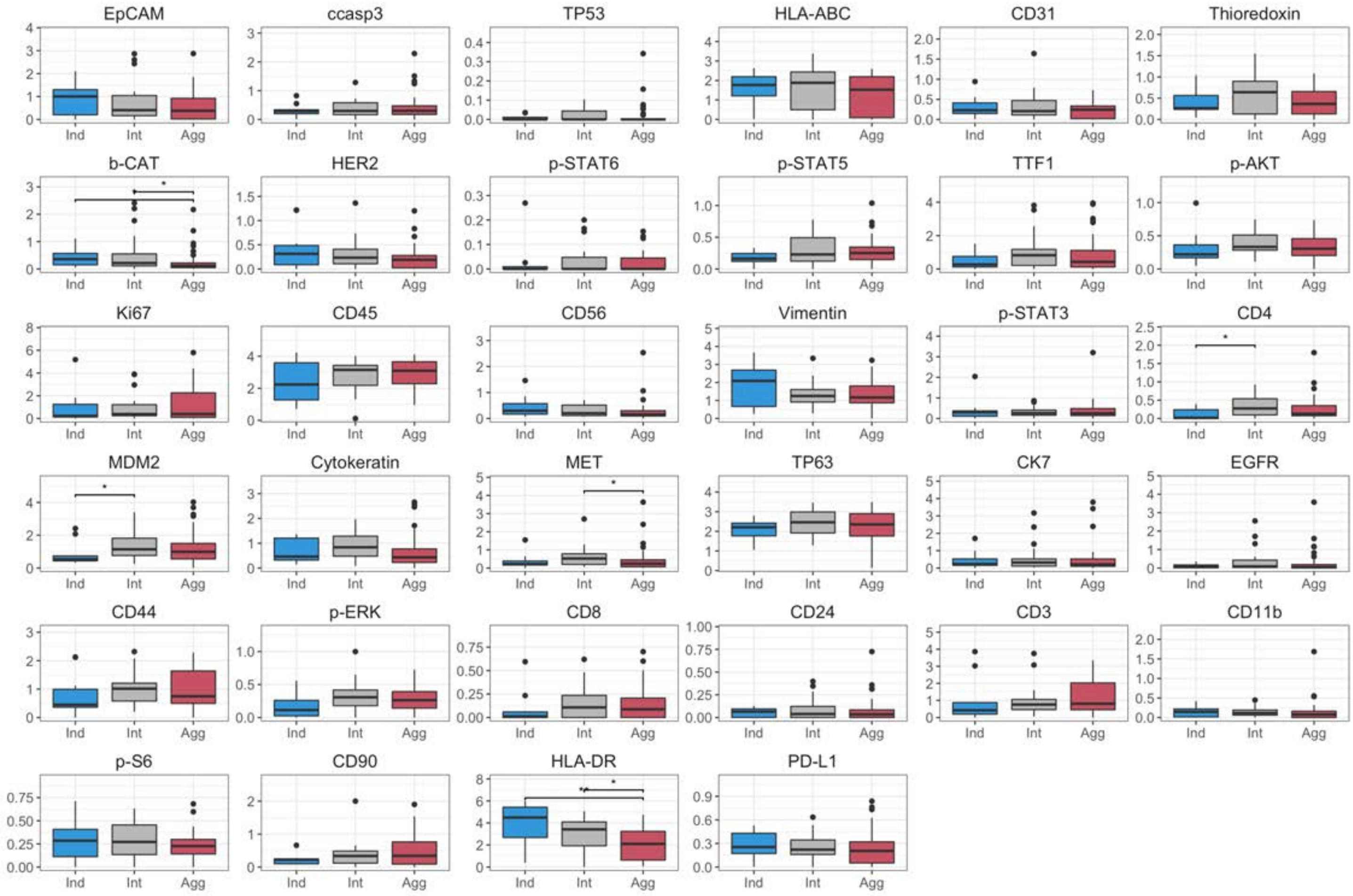

Figure S11. Differential bulk protein expression analysis per patient sample. No star=pvalue>0.05, *=pvalue<0.05, **=pvalue<0.001.

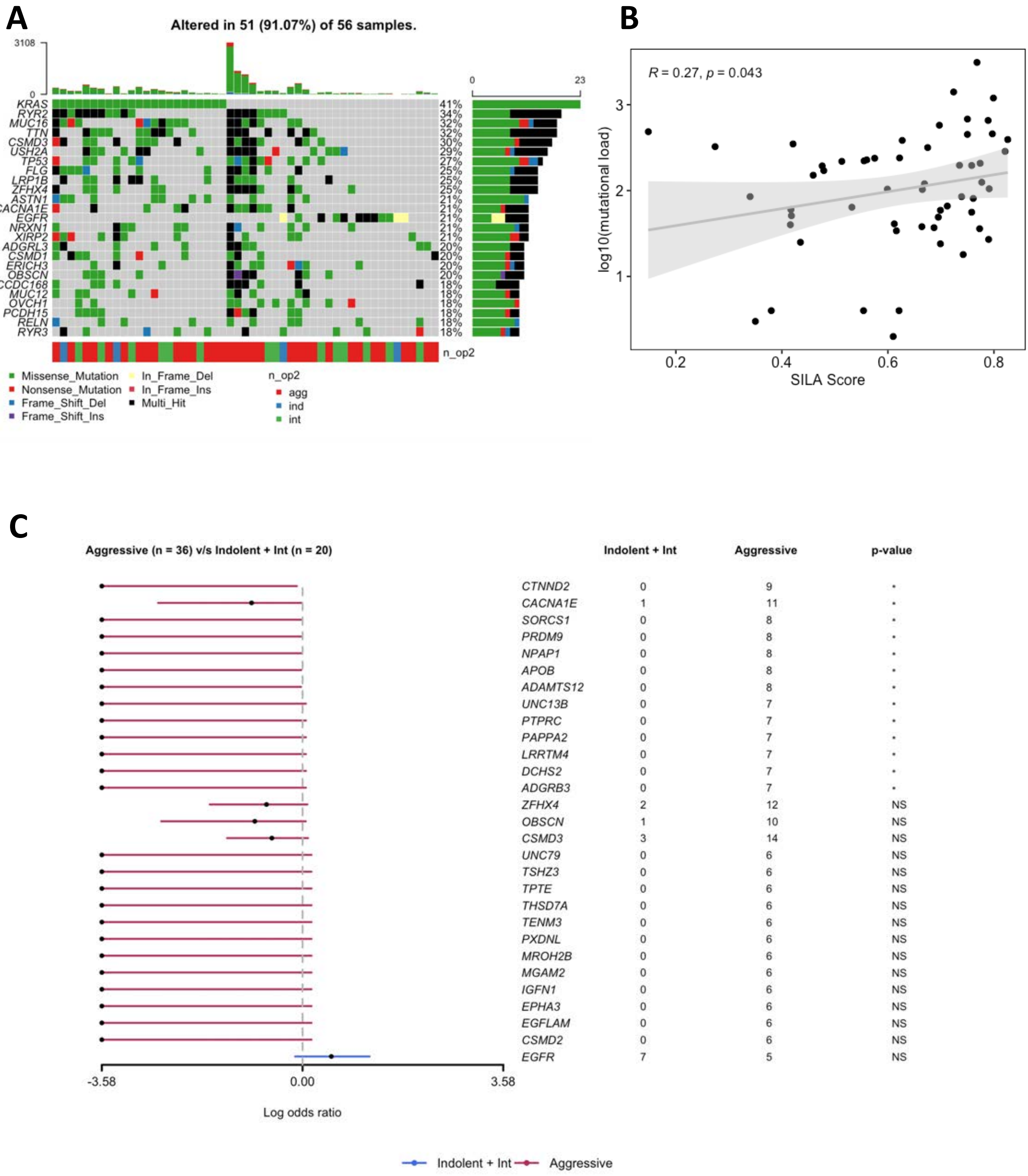

Figure S12. Whole Exome Sequencing data analysis. (A) Oncoplot showing top 25 mutated genes. (B) Spearman correlation of SILA score and Log10 of mutational load per patient. (C) Clinical enrichment analysis of mutations comparing Indolent+Intermediate versus Aggressive tumor samples.

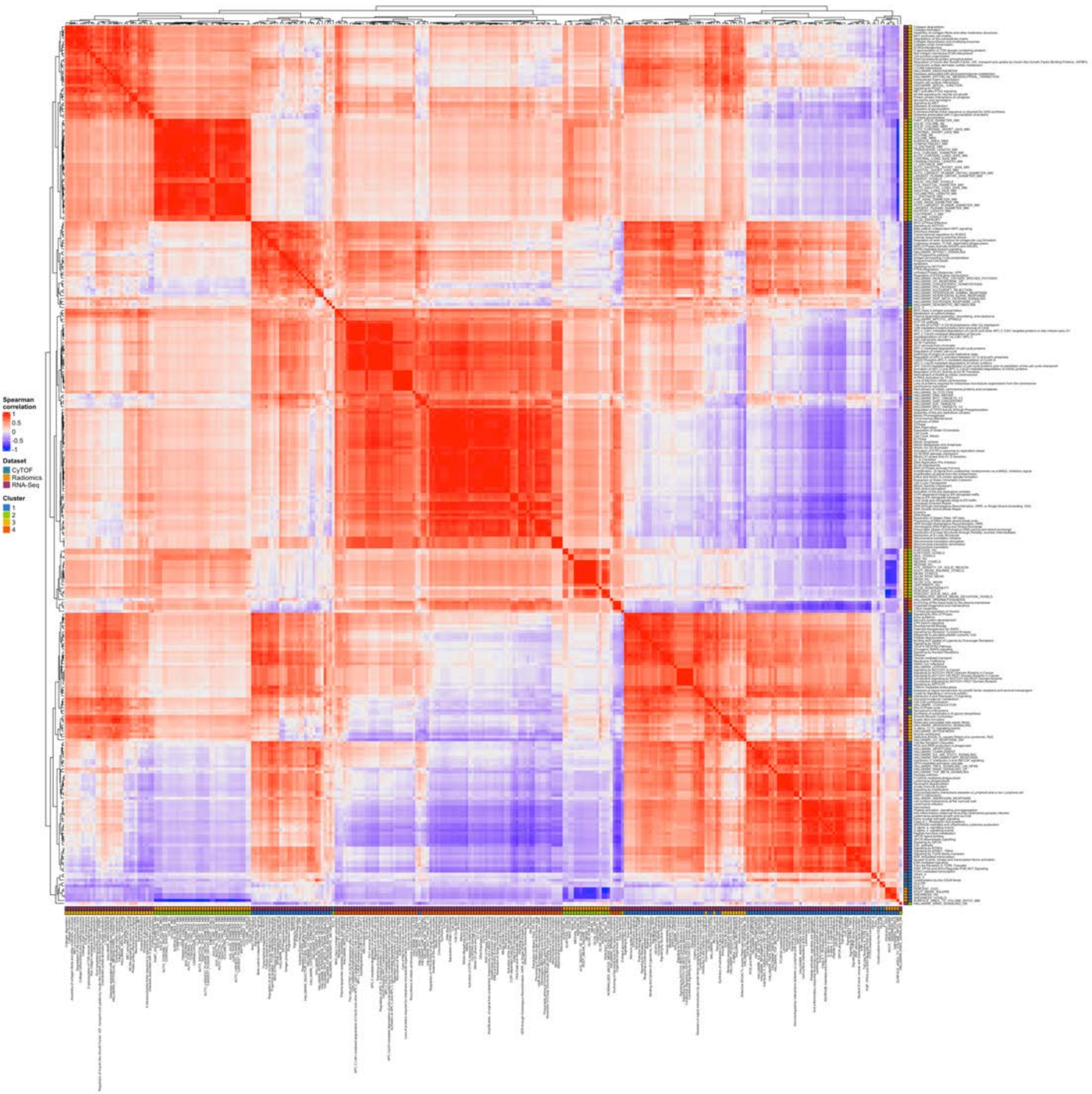

Figure S13. Similarity matrix of features used for data integration.

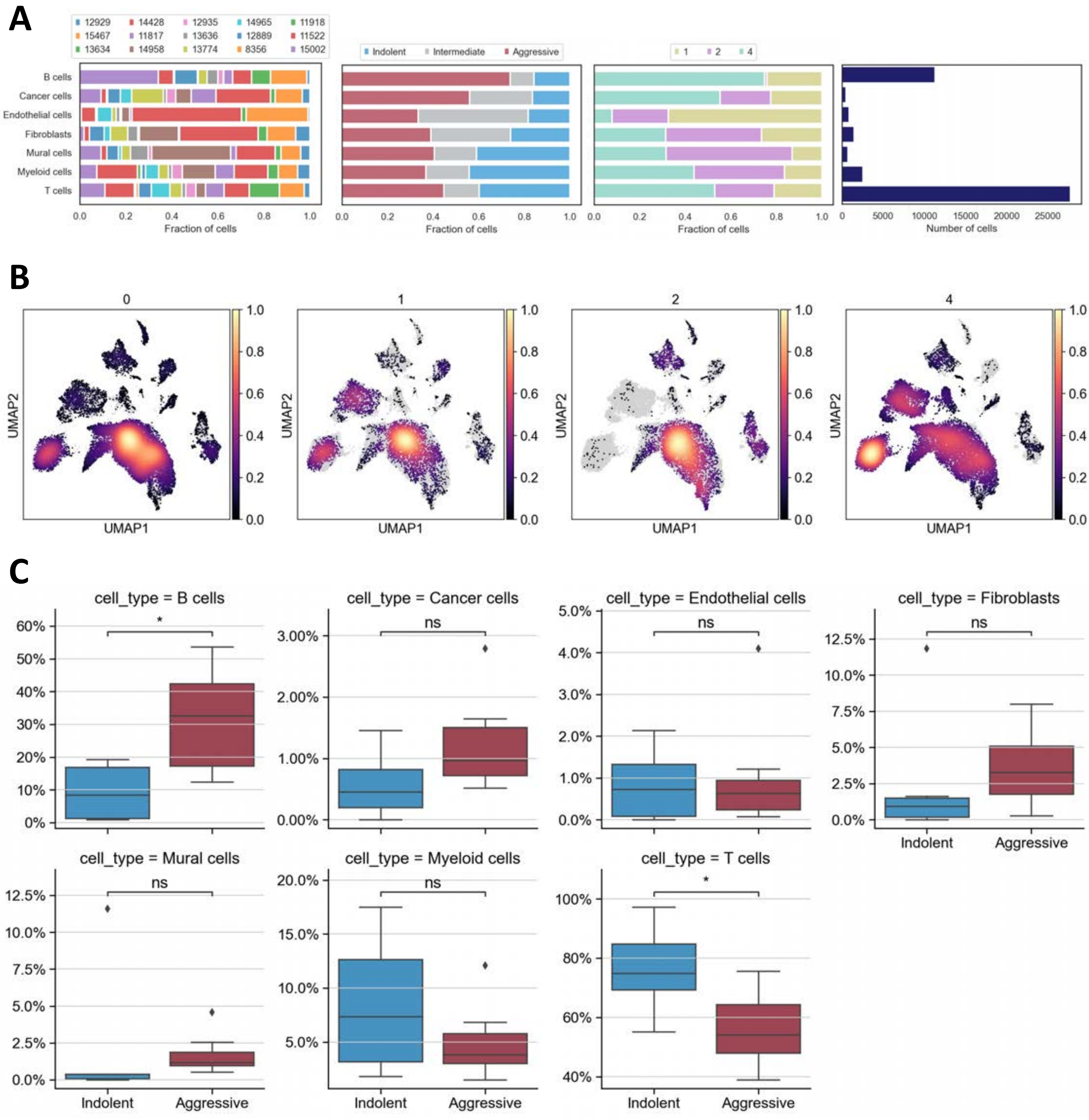

Figure S14. Single cell RNA-Seq analysis of 15 tumor samples. (A) Fraction of cells per cell type colored by patient ID, risk group, data integration patient cluster, and number of cells per cell type. (B) UMAP representation of 44867 cells from 15 patients colored by cell density. Labels correspond to data integration patient cluster 1=P1, 2=P2, 4=P4, 0=patients not included in data integration. (C) Differential abundance analysis. Y axis corresponds to the fraction of cells per patient sample. ns=pvalue>0.05, *=pvalue<0.05, **=pvalue<0.001.

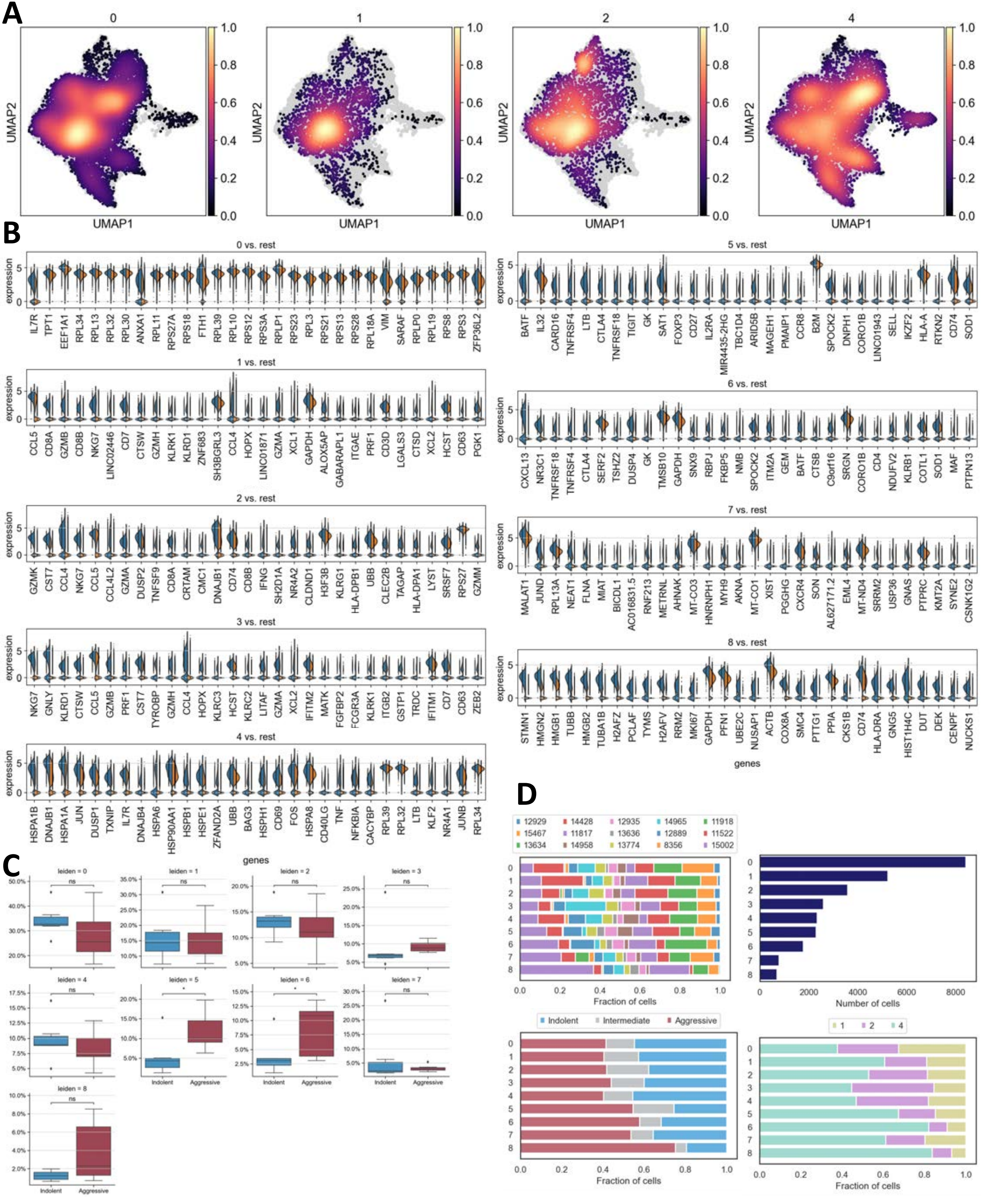

Figure S15. T cells cluster analysis. (A) UMAP representation of 27708 cells from 15 patients colored by cell density. Labels correspond to data integration patient cluster 1=P1, 2=P2, 4=P4, 0=patients not included in data integration. (B) Split violin visualization showing the top 30 marker genes for each cluster when compared to the rest.. (C) Differential abundance analysis. Y axis corresponds to the fraction of cells per patient sample. ns=pvalue>0.05, *=pvalue<0.05, **=pvalue<0.001. (D) Fraction of cells per cluster colored by patient ID, risk group, data integration patient cluster, and number of cells per cluster.

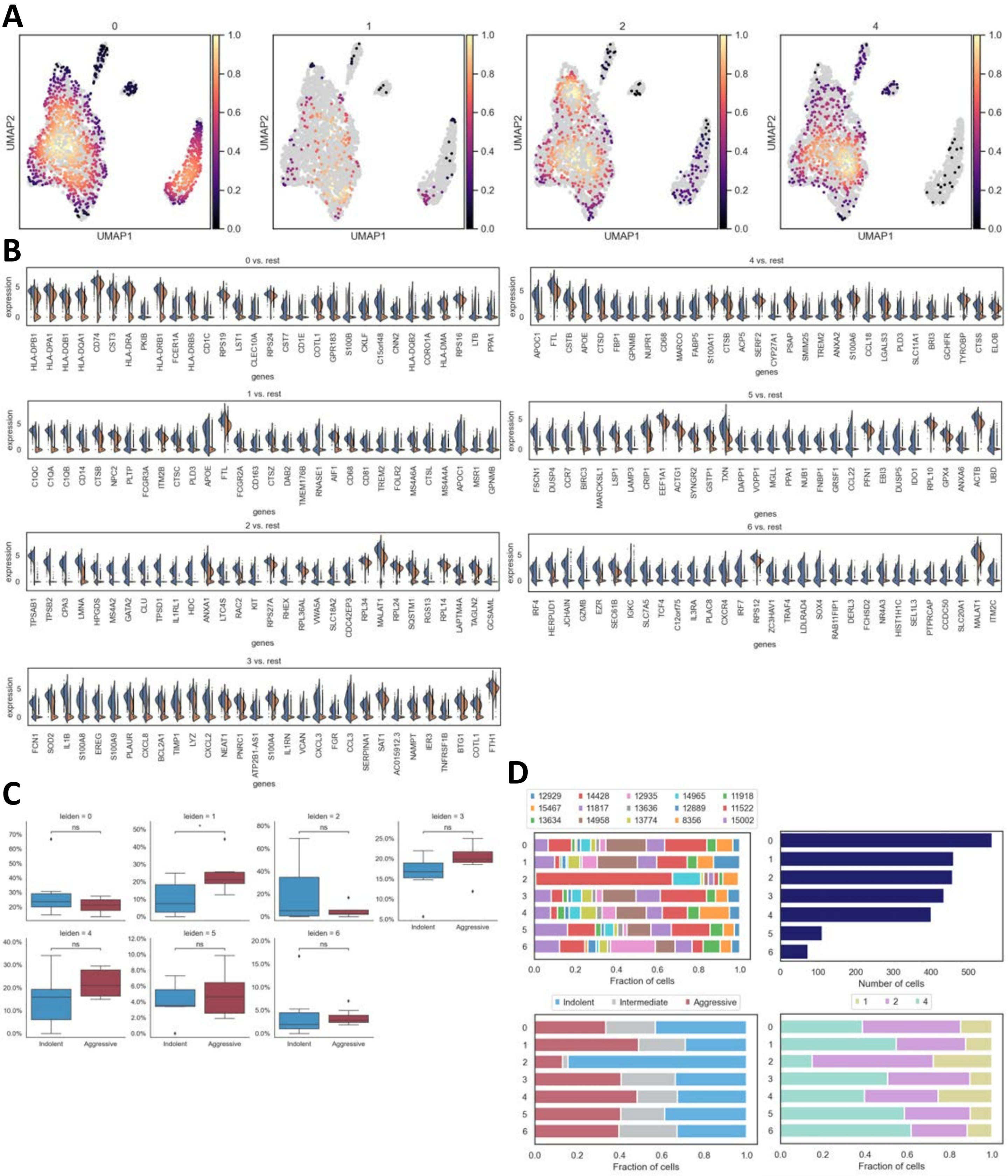

Figure S16. Myeloid cells cluster analysis. (A) UMAP representation of 2497 cells from 15 patients colored by cell density. Labels correspond to data integration patient cluster 1=P1, 2=P2, 4=P4, 0=patients not included in data integration. (B) Split violin visualization showing the top 30 marker genes for each cluster when compared to the rest.. (C) Differential abundance analysis. Y axis corresponds to the fraction of cells per patient sample. ns=pvalue>0.05, *=pvalue<0.05, **=pvalue<0.001. (D) Fraction of cells per cluster colored by patient ID, risk group, data integration patient cluster, and number of cells per cluster.

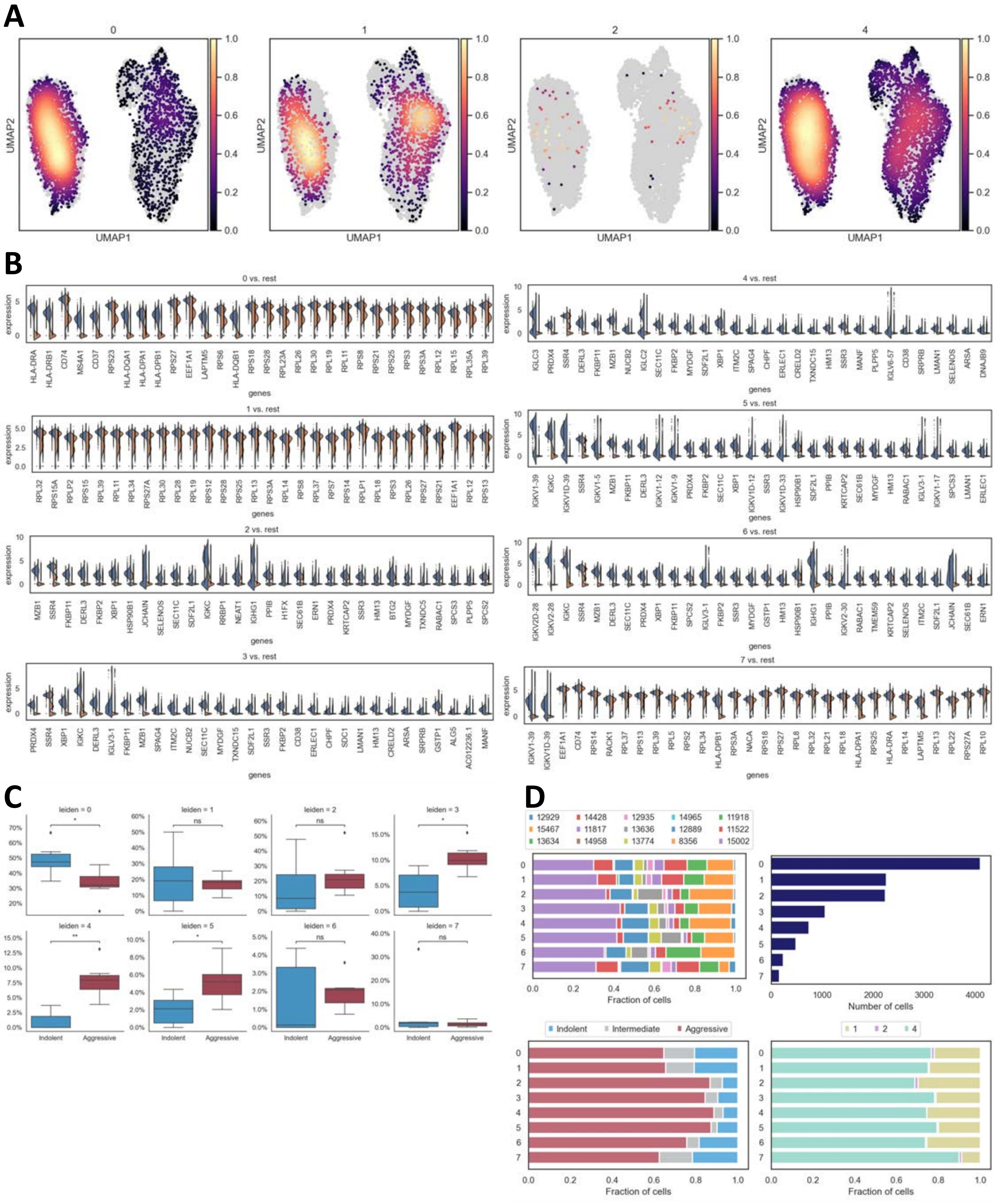

Figure S17. B cells cluster analysis. (A) UMAP representation of 11246 cells from 15 patients colored by cell density. Labels correspond to data integration patient cluster 1=P1, 2=P2, 4=P4, 0=patients not included in data integration. (B) Split violin visualization showing the top 30 marker genes for each cluster when compared to the rest.. (C) Differential abundance analysis. Y axis corresponds to the fraction of cells per patient sample. ns=pvalue>0.05, *=pvalue<0.05, **=pvalue<0.001. (D) Fraction of cells per cluster colored by patient ID, risk group, data integration patient cluster, and number of cells per cluster.

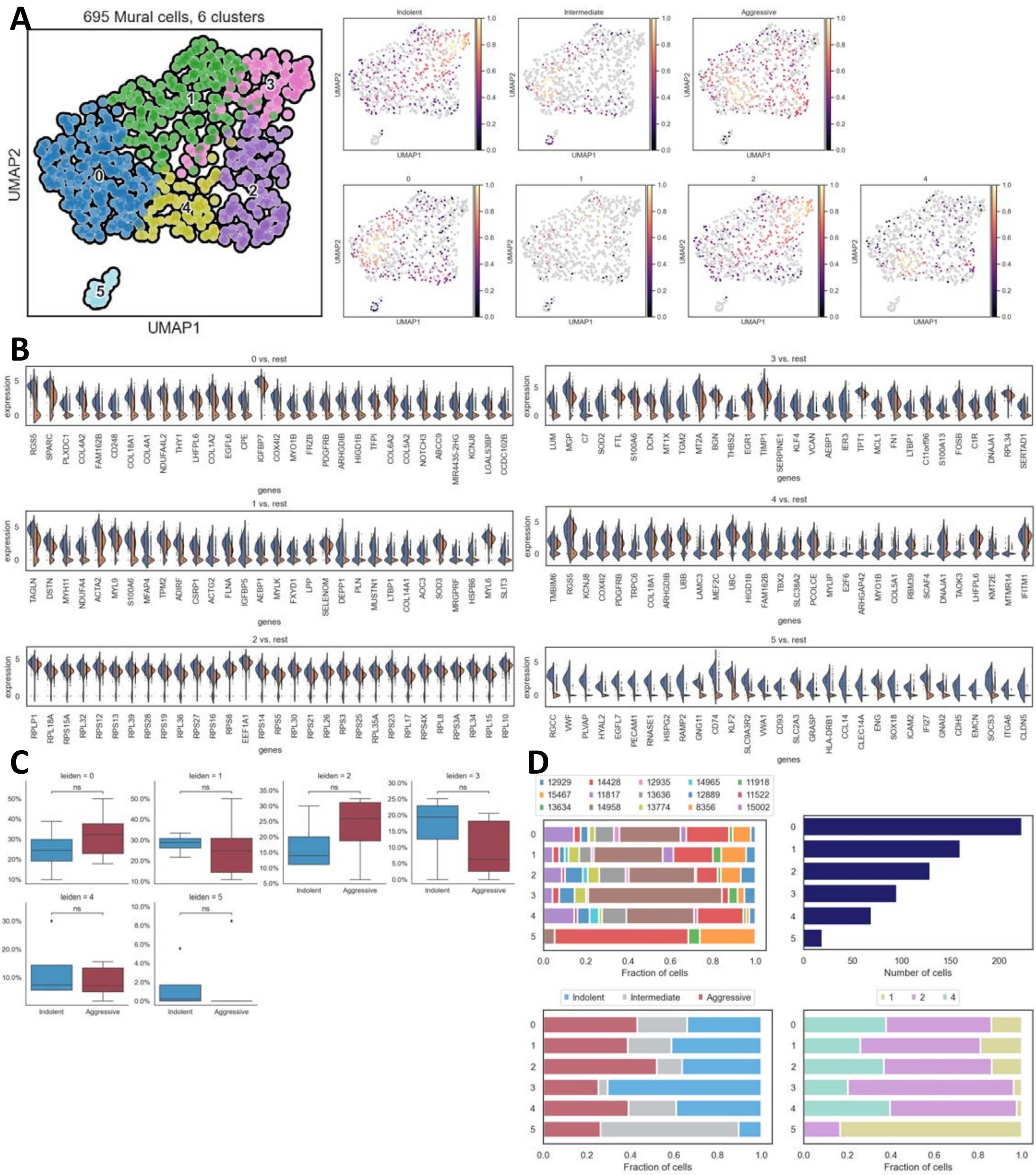

Figure S18. Mural cells cluster analysis. (A) UMAP representation of 695 cells from 15 patients colored by cluster identity, and cell density devided by risk group (up) or data integration patient clusters (down) (1=P1, 2=P2, 4=P4, 0=patients not included in data integration). (B) Split violin visualization showing the top 30 marker genes for each cluster when compared to the rest.. (C) Differential abundance analysis. Y axis corresponds to the fraction of cells per patient sample. ns=pvalue>0.05, *=pvalue<0.05, **=pvalue<0.001. (D) Fraction of cells per cluster colored by patient ID, risk group, data integration patient cluster, and number of cells per cluster.

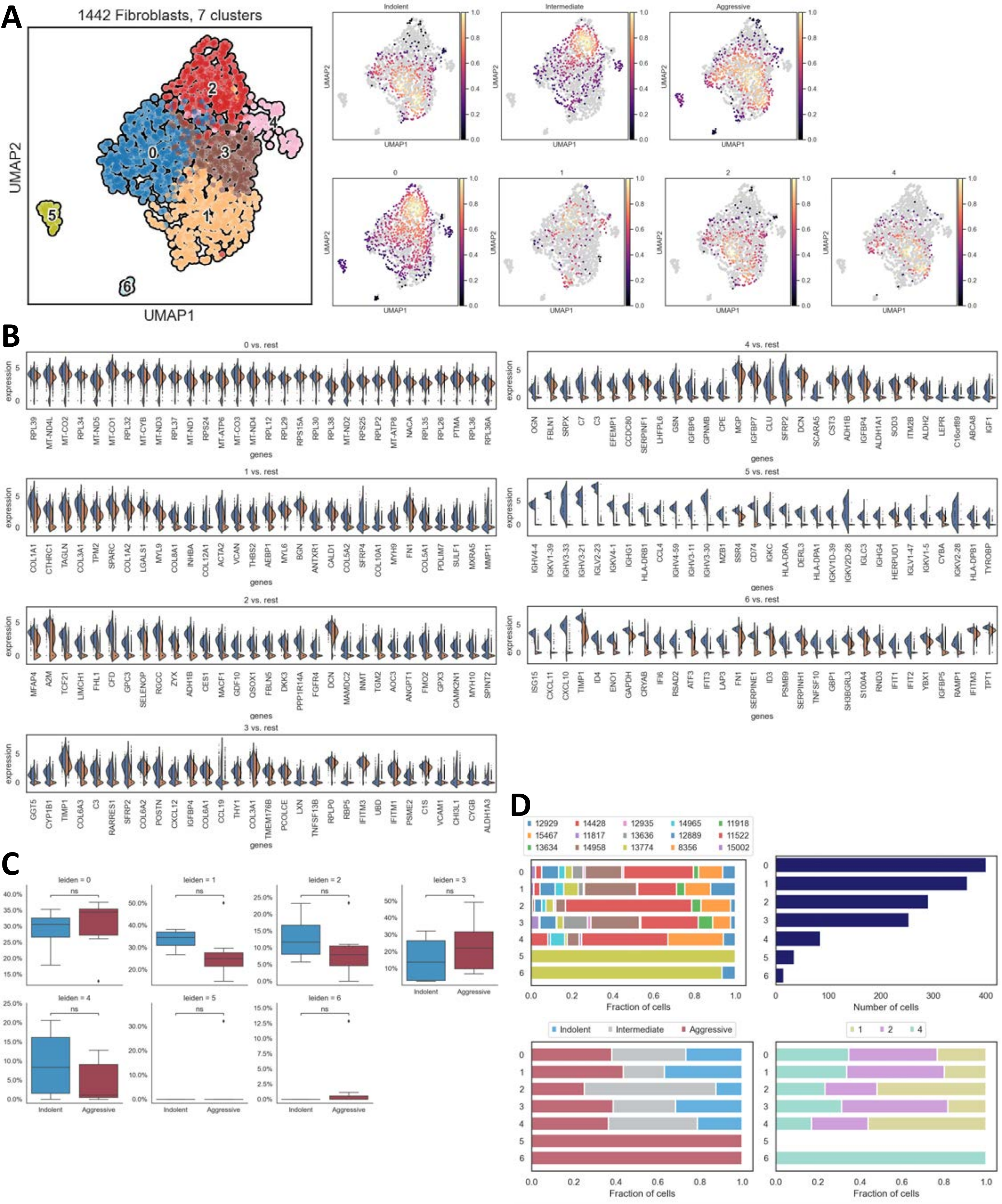

Figure S19. Fibroblasts cells cluster analysis. (A) UMAP representation of 1442 cells from 15 patients colored by cluster identity, and cell density devided by risk group (up) or data integration patient clusters (down) (1=P1, 2=P2, 4=P4, 0=patients not included in data integration). (B) Split violin visualization showing the top 30 marker genes for each cluster when compared to the rest.. (C) Differential abundance analysis. Y axis corresponds to the fraction of cells per patient sample. ns=pvalue>0.05, *=pvalue<0.05, **=pvalue<0.001. (D) Fraction of cells per cluster colored by patient ID, risk group, data integration patient cluster, and number of cells per cluster.

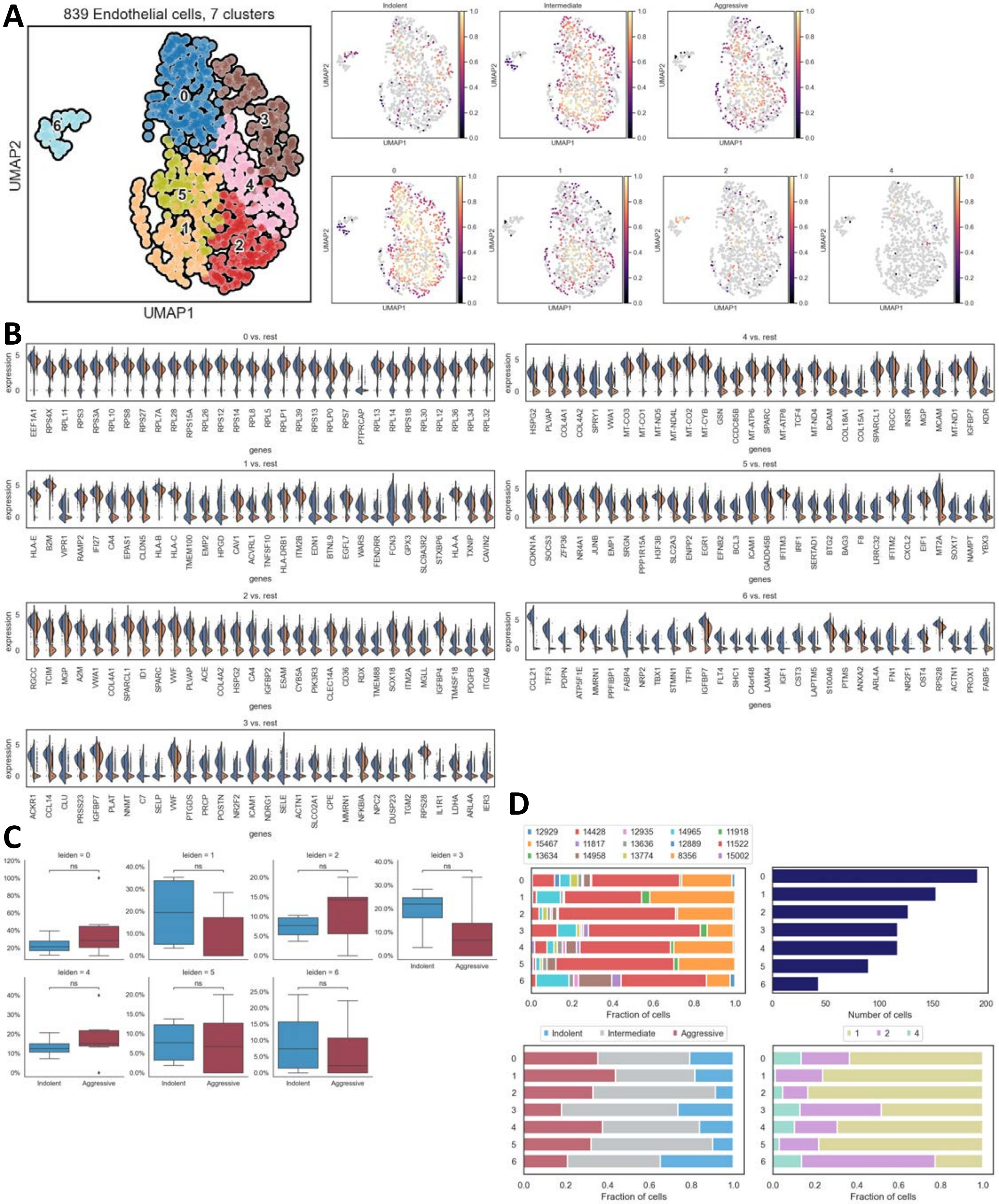

Figure S20. Endothelial cells cluster analysis. (A) UMAP representation of 839 cells from 15 patients colored by cluster identity, and cell density devided by risk group (up) or data integration patient clusters (down) (1=P1, 2=P2, 4=P4, 0=patients not included in data integration). (B) Split violin visualization showing the top 30 marker genes for each cluster when compared to the rest.. (C) Differential abundance analysis. Y axis corresponds to the fraction of cells per patient sample. ns=pvalue>0.05, *=pvalue<0.05, **=pvalue<0.001. (D) Fraction of cells per cluster colored by patient ID, risk group, data integration patient cluster, and number of cells per cluster.

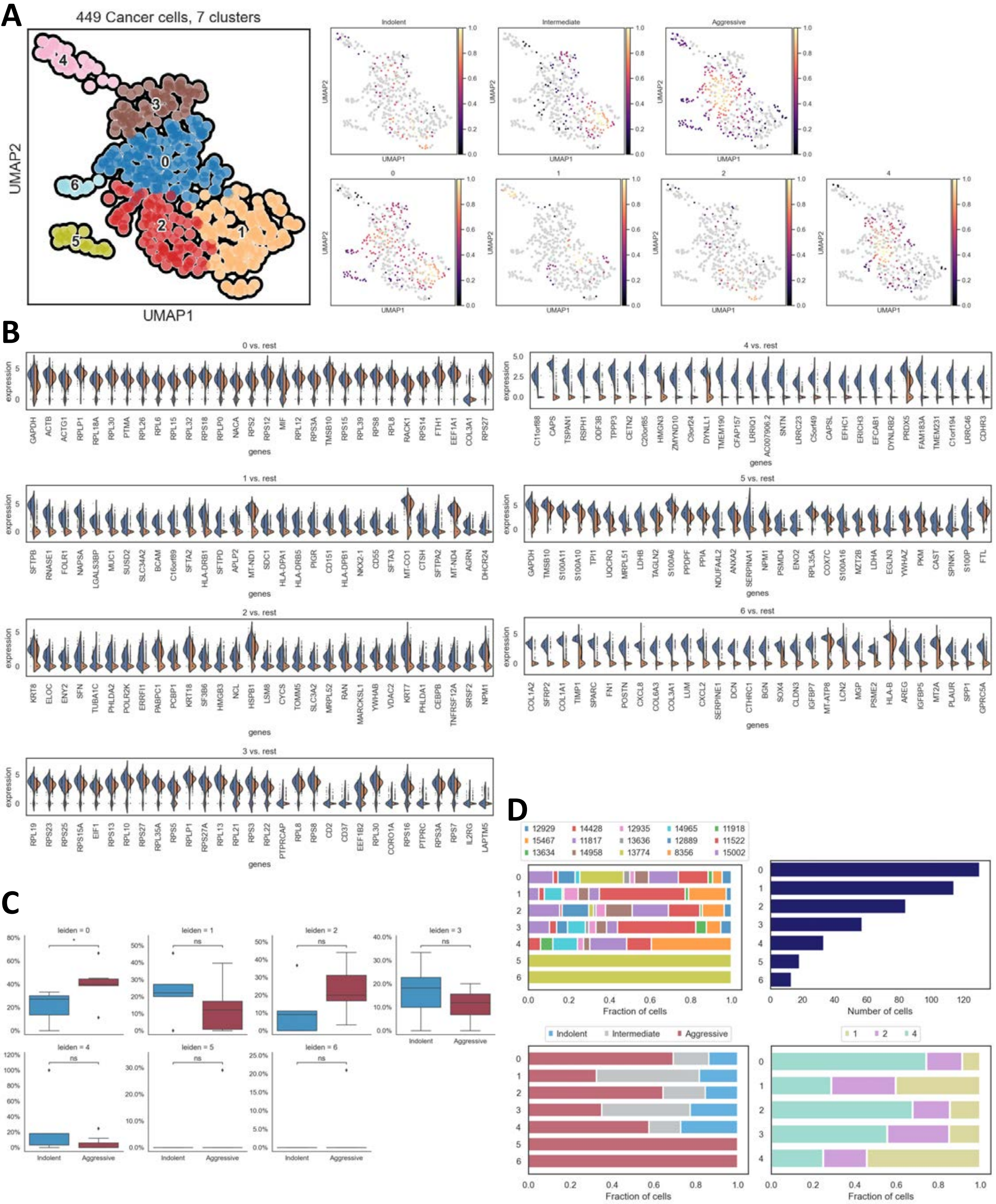

Figure S21. Cancer cells cluster analysis. (A) UMAP representation of 449 cells from 15 patients colored by cluster identity, and cell density devided by risk group (up) or data integration patient clusters (down) (1=P1, 2=P2, 4=P4, 0=patients not included in data integration). (B) Split violin visualization showing the top 30 marker genes for each cluster when compared to the rest.. (C) Differential abundance analysis. Y axis corresponds to the fraction of cells per patient sample. ns=pvalue>0.05, *=pvalue<0.05, **=pvalue<0.001. (D) Fraction of cells per cluster colored by patient ID, risk group, data integration patient cluster, and number of cells per cluster.
